# Neuronal heterogeneity and stereotyped connectivity in the auditory afferent system

**DOI:** 10.1038/s41467-018-06033-3

**Published:** 2018-09-12

**Authors:** Charles Petitpré, Haohao Wu, Anil Sharma, Anna Tokarska, Paula Fontanet, Yiqiao Wang, Françoise Helmbacher, Kevin Yackle, Gilad Silberberg, Saida Hadjab, François Lallemend

**Affiliations:** 1 Department of Neuroscience, Karolinska Institutet, Biomedicum, Stockholm, 171 77 Sweden; 2 Aix-Marseille Université, CNRS UMR7288, Institut de Biologie du Développement de Marseille (IBDM), 13009, Marseille, France; 3 Department of Physiology, University of California–San Francisco, San Francisco, CA 94158 USA

## Abstract

Spiral ganglion (SG) neurons of the cochlea convey all auditory inputs to the brain, yet the cellular and molecular complexity necessary to decode the various acoustic features in the SG has remained unresolved. Using single-cell RNA sequencing, we identify four types of SG neurons, including three novel subclasses of type I neurons and the type II neurons, and provide a comprehensive genetic framework that define their potential synaptic communication patterns. The connectivity patterns of the three subclasses of type I neurons with inner hair cells and their electrophysiological profiles suggest that they represent the intensity-coding properties of auditory afferents. Moreover, neuron type specification is already established at birth, indicating a neuronal diversification process independent of neuronal activity. Thus, this work provides a transcriptional catalog of neuron types in the cochlea, which serves as a valuable resource for dissecting cell-type-specific functions of dedicated afferents in auditory perception and in hearing disorders.

## Abstract (web summary)

Spiral ganglion neurons (SGNs) of the cochlea receive input from hair cells and project to the auditory brainstem. Here, the authors perform single-cell RNA sequencing to identify four SGN subclasses and characterize their molecular profile, electrophysiological properties and connectivity.

## Introduction

The perception of sound is essential to receive information from our environment, and to communicate and interact socially. Hair cells (HCs) in the cochlea transduce sound and convey its signal to the central nervous system via chemical synapses on the spiral ganglion (SG) neurons dendrites^1,2^. The central afferents of these SG neurons converge to form the auditory nerve, which connects to the cochlear nuclei in the brainstem. The auditory nerve is the sole supply route of auditory information from HCs to the brain, and contained processed information about sound frequency, intensity, timbre, and pitch which are all necessary for perceptual sound detections, discriminations, and recognitions centrally^3–5^. However, the cellular basis of the processing and routing of these auditory qualities at the periphery are still poorly understood.

Processing of the sound signal in the auditory nerve has been hypothesized to originate in the diversity of biophysical properties of type I SG neuron fibers (95% of auditory afferents). For instance, frequency specific stimulus activation of specific groups of afferents has been shown to reflect the contribution of different SG fibers with distinct temporal discharge patterns^3,6,7^. Another example of auditory fibers diversity is the intensity driven activation of selective auditory afferents^5,8,9^, where at least two populations of auditory fibers can be distinguished on the basis of their threshold activity: the low threshold (LT) fibers and the high threshold (HT) fibers. Additionally, HT fibers have wide range of sensitivity to sound levels which has been suggested to encode the enormous range of intensities in the auditory system^5,8^. Thus, since cochlear transduction depends on an interaction between mechanical processes but also the electrical properties of auditory afferents, we need to understand how these, which are foundational for the auditory experience, contribute more specifically to decode the various features of the incoming sound and how their dysfunction may lead to neural hearing impairments.

To further unravel the mechanisms of sound processing in the peripheral auditory system, we used single-cell RNA sequencing (scRNAseq) combined with genetic labeling to fully uncover the molecular types of SG neurons. We identified four types of neurons, including three novel subclasses of type I neurons and the type II neurons, along with numerous new marker genes and provided a comprehensive genetic framework that may shape their synaptic communication patterns. Second, using newly identified markers, we characterized the differential projection patterns of the distinct subclasses of type I neurons to the inner hair cells (IHCs), the actual sensory receptors, and recorded their electrophysiological properties. Finally, a similar analysis on developing SG neurons provided evidence of their perinatal diversification, before the onset of hearing, as well as distinctive expression patterns of key signaling pathway components predictive of this functional diversification. More generally, our study unveils a large molecular heterogeneity in the cochlear afferent system that delineates previously uncharacterized neuron types, which likely represent discrete ascending channels that convey distinct auditory information. In addition, it also constitutes a toolbox to develop genetic approaches to examine the function of the different SG neuron types in hearing.

## Results

### scRNAseq identifies new neuron types in the cochlea

The majority of SG neurons are type I neurons; they are myelinated, contact the inner hair cells (IHCs) and are the principal carrier of the auditory signal. The other minority population (5%) of neurons are called type II neurons; they are unmyelinated and innervate the outer hair cells (OHCs), which modulate the output of the organ of Corti^1^. The few in vitro studies on their function indicate they could report cochlear trauma^10,11^.

**Fig. 1:**
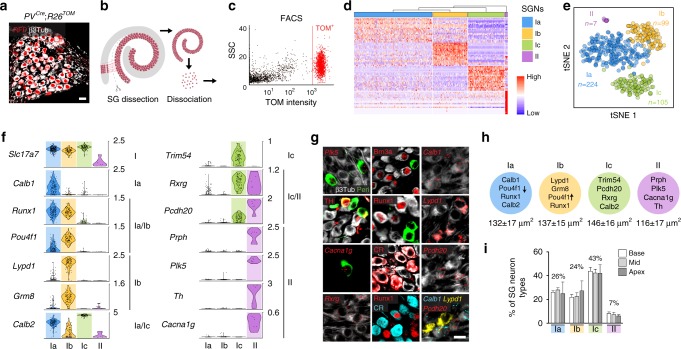
Identification and validation of four neuronal types in adult SG. **a** Genetic tracing of SG neurons (β3Tub^+^/RFP^+^) on P21 sections from *PV*^*cre*^;*R26*^*TOM*^ mice. **b** Sketch depicting dissection of SG from the organ of Corti and their dissociation. **c** Fluorescence-side scatter plot of dissociated single cells showing isolation of Tom^+^ SG neurons through FAC sorting. **d** Heat map showing single-cell expression of the top 20 differentially expressed genes in the four types of SG neurons, from combined data of P17, P21, and P33 neurons (SGNs, SG neurons). Dendritic tree shows the similarity between neuronal types. **e** tSNE plot showing four distinct types of SG neurons. **f** Violin plots showing the expression of marker genes in log-transformed scale among the four different populations of SG neurons. **g** In vivo validation of the identified SG neuron types by immunohistochemical and fluorescent in situ hybridization using identified marker genes in P21 cochlea. Type II neurons were identified by peripherin (Peri), *Plk5*, TH, and *Cacna1g* specifically. Ia neurons were identified by *Calb1*, *Pou4f1*, Runx1, and calretinin (CR). Ib neurons were identified by *Lypd1*, Runx1, and *Pou4f1* and Ic neurons, by *Rxrg*, *Pcdh20*, and CR expression. Note that co-localization on sections could never be observed for markers expressed in different populations of neurons in the scRNAseq data. **h** Schematic representation of neuronal types with their key markers and their average soma size (in µm^2^) at P21. **i** Proportion of SG neurons types along the tonotopic gradient (from base to apex) quantified by Runx1, Peri, and CR expression at P21 (*n* = 3 animals; Data are represented as mean ± SEM). Scale bars: 20 μm (**a**,**g**)

To identify neuron types in adult SG neurons, a total of 487 tdTomato positive (TOM^+^) cells from *PV*^*Cre*^;*R26*^*TOM*^ cochlea of postnatal stage 17 (P17), P21, and P33 were processed for single-cell transcriptome analysis (Fig. 1a–c). Note that all SG neurons express parvalbumin (PV) and are TOM^+^ after recombination (Fig. 1a). The cell expression data were clustered using R package SEURAT and visualized using bi-dimensional t-distributed stochastic neighbor embedding (t-SNE), showing four distinct neuronal types (Fig. 1d–e). The type II neurons represented the smallest cluster and were identified by their expression of the known markers *Prph*^12^ (peripherin) and *Th*^13^ (Fig. 1e–f). We confirmed the type I identity of the three other clusters (thereafter named Ia, Ib, and Ic types) by their high expression of the transcription factor *Prox1*^14^ and lack of *Prph* (Fig. 1e, Supplementary Fig. 1a). Importantly, our initial clustering showed that these neuron type identities were conserved at P17, P21, and P33 (Supplementary Fig. 1b).

We next explored this data set to identify novel markers of the four types of SG neurons (Fig. 1f, Supplementary Fig 1c; Supplementary Data 1, 2). All type I neurons (Ia, Ib, and Ic) were characterized by the expression of *Slc17a6* (VGlut1), *Trpm2*, *Epha4,* and abundant levels of *Prox1*, and the type II population, by its expression of *Prph*, *Npy* (neuropeptide Y), *Th*, *Piezo2,* and high levels of *Cux2*. The different subclasses of type I neurons could be further distinguished based on unique and combinatorial molecular profiles. Type Ia expressed *Runx1*, *Ttn* (Titin), *Calb1* and *2* (calretinin, CR), and low levels of *Pou4f1* (Brn3a). Type Ib was characterized by the expression of *Grm8* (mGluR8), *Kcnc2* (K_v_3.2), *Lypd1*, *Runx1*, and abundant levels of *Pou4f1*. Finally, type Ic expressed *Trim54* (MuRF3), *Rxrg* and high levels of *Calb2*. We next confirmed in vivo the existence of the four types of SG neurons by validating the expression of genes enriched in specific cell types using combinatorial labeling with new markers and transgenic mouse lines (Fig. 1g). In addition, we analyzed the soma size of each neuronal type (Fig. 1h) and calculated the percentage of each type along the baso-apical axis of the cochlea, which physiologically reflects the tonotopic gradient necessary for encoding frequency specificity within the auditory nerve^8,15^. Using different combinations of newly identified specific markers, the proportion of the four neuron types was found to be relatively constant throughout the length of the cochlea: 7% for type II, 26% for type Ia, 24% for type Ib, and 43% for type Ic (Fig. 1i, Supplementary Fig. 1d-f). Also, we could not observe any specific spatial patterning within the SG of any of the type I neurons subclasses (Supplementary Fig. 1f-g).

Together, these results demonstrate the existence and validate markers of four types of SG neurons. We investigate below the transcriptional basis that contributes to define their distinct neuronal identity.

### Comparative analysis of SG neurons

**Fig. 2:**
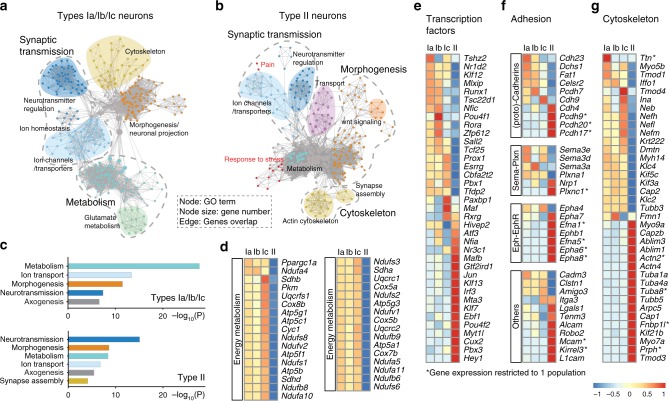
Comparative analysis of SG neurons transcriptomes. **a**, **b** Gene set enrichment analysis of types I (**a**) and type II neurons (**b**) visualized by network. Each node represents a GO term, edges are drawn when there are shared genes between two GO terms. **c** Gene ontology analysis of the type I and type II group. The graph shows most significant terms reflecting neuronal features. **d** Heatmaps showing expression of genes associated with energy metabolism in each subclass of SG neurons. **e**–**g** Differential expression of transcription factors (**e**), cell-adhesion molecules including Cadherin, Semaphorin, and Ephrin family (**f**) and of cytoskeleton-related genes among the four subclasses of SG neurons (**g**) (see also Supplementary Fig. 2)

To get insights into the major differences between the four types of SG neurons, we first conducted gene set enrichment analysis (GSEA) of the two most distinct populations, i.e., type I and type II neurons. While highly enriched gene ontology (GO) terms were associated with neuronal cell functions, such as “neurotransmission”, “ion transport”, and “axogenesis”, the most enriched category in the type I group was “metabolism” (Fig. 2a, c, d), likely reflecting the large energy demand of the myelinated type I neurons to ensure their high sensitivity and temporal fidelity^16,17^. Also, this analysis identified an enrichment of genes among the unmyelinated type II neurons involved in “response to stress” and “pain” mechanisms (Fig. 2b), which is consistent with their activation by cochlear damage^11^.

To further complete the characterization of the four types of SG neurons, we carried out a comparative analysis of their differential expression of transcription factors, adhesion, and cytoskeleton molecules. While numerous genes were specifically expressed in type II neurons, a strict ON/OFF division amongst the three subclasses of type I neurons was rarely observed (Fig. 2e–g, Supplementary Fig. 2), likely reflecting the importance of a combinatorial basis of the code specifying a cell type.

### Neurotransmission-related machinery in SG neurons

**Fig. 3:**
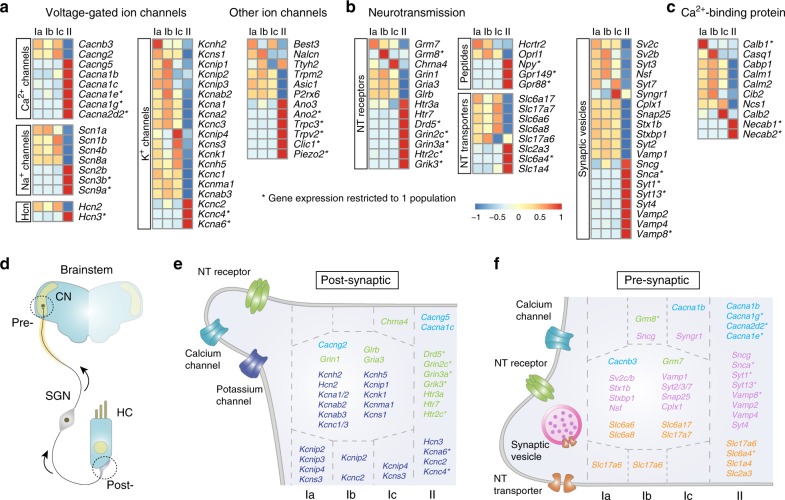
Input–output communication transcriptional signature of SG neurons. **a** Differential expression of voltage-gated ion channels family among SG neurons. **b**, **c** Differential expression of neurotransmission systems including neurotransmitter (NT) receptors, peptides-related molecules, NT transporters, and synaptic vesicles (**b**) and of calcium-binding protein (**c**) among SG neurons (see also Supplementary Fig. 3). **d** Sketch of the SG neuron (SGN) synaptic connection with HC peripherally and with the cochlear nuclei (CN) in the brainstem. Arrows shows direction of the signal transmission. **e**, **f** Schematic representation of the transcriptional portrait of the post-synaptic (**e**) and pre-synaptic (**f**) sites of SG neuron types, based on differentially expressed gene shown in **a**, **b**

We next analyzed in more detail gene families implicated in generic neuronal transmission, including voltage-gated ion channels (VGICs), synaptic vesicle complex, neurotransmitter (NT) receptors and transporters, and calcium-binding proteins, as they all participate in the classification of neuron types by regulating their electrophysiological profile (Fig. 3a–c). SG neurons respond mainly to glutamate released by HCs^16,18^. Interestingly, the relative expression of ionotropic glutamate receptors (iGluR)—AMPA (*Gria*), Kainate (*Grik*), and N-methyl-d-aspartate (NMDA) (*Grin*) receptors—and of metabotropic glutamate receptors (mGluRs, *Grm*) was particularly heterogeneous between the type I and type II neurons. Only type I neurons were found to express mGluRs (*Grm7* in all three type I, and *Grm8* only in type Ib) which are mostly pre-synaptic and decrease neurotransmitter release at the central synapse^19^ (Fig. 3a–f, Supplementary Figs. 3 and 4a,b). Also, while type I neurons exhibited a homogeneous pattern of iGluR expression, *Gria3* and *Grin1* were expressed at much lower levels in type II neurons than in all other types, whereas type II neurons instead specifically expressed *Grik3*, *Grin2c*, and *3a*. *Grik4* and *5* were detected in all subtypes, albeit at very low levels. These results suggest that type II neurons use both AMPA and Kainate receptors, while type I neurons rely mainly on AMPA receptors, as previously shown^16,18^. Moreover, the distinct composition of NMDARs subunits suggests cell-type-dependent activation of different sets of NMDARs-interacting molecules^20^. Thus, together with the morphological differences of the OHC-type II afferent synapse, the different expression of iGluR by type II neurons could participate in vivo in establishing their distinct synaptic responses; the frequency of synaptic events and the size of synaptic potentials are considerably smaller in type II afferent dendrites compared to type I afferents^10,21^.

The electrophysiological properties of neurons are shaped by the expression of several families of VGICs, which include the sodium (Na_v_), potassium (K_v_), calcium (Ca_v_) channels, and the hyperpolarization-activated cyclic nucleotide-gated (HCN) channels^22^. VGICs exhibited extensive differential expression profiles amongst the different types of SG neurons, sharply distinguishing type II from type I neurons (Fig. 3a–f). Although Ca_v_, Na_v_, and HCN channels families showed modest differences amongst the different subclasses of type I neurons, K_v_ channels differed substantially in these neuron types. Activation of K_v_ channels shapes the pattern of neuronal firing and excitability and drives the rate of adaptation to sustained stimuli^23^. Their differential expression could participate in the expected distinct physiological characteristics of the different subclasses of type I neurons^5,8,24^ (Fig. 3d–f and Supplementary Fig. 4a and b).

The excitability of auditory afferents is also regulated by the lateral and medial olivocochlear (LOC and MOC) efferents which innervate respectively the type I and type II peripheral endings underneath the HCs and modulate their sensitivity^1,2,25^. Note that the MOC efferents make also direct synaptic contacts with OHCs. The MOC system is cholinergic and GABAergic while the LOC system can be divided into at least cholinergic and dopaminergic components and also contain serotonin, GABA and many neuropeptides, including enkephalins, dynorphins, and calcitonin gene-related peptide (CGRP)^25,26^. Surprisingly, the receptors for these peptides were absent from all SG neurons, as were dopamine receptors, with the exception of *Drd5* that was expressed in type II neurons, and *Drd1a* in type Ib neurons, albeit both at low levels (Supplementary Fig. 4c). The ionotropic GABA_A_ receptor subunit *Gabrb3* and metabotropic receptor *Gabbr1/2* (GABA_B1/2_) were uniformly expressed amongst SG neurons. In contrast, nicotinic acetylcholine receptors (nAChRs, which are ionotropic) were differentially expressed: *Chrnb2* in all SG neurons, *Chrna7* in type II and *Chrna4* in type Ic, all at low levels in general. Interestingly, only the type II neurons expressed serotonin receptors (mostly *Htr2c* and *7*) (Fig. 3b; Supplementary Fig. 4c). Thus, with regards to the very low abundance of receptors for acetylcholine (Ach), dopamine, and opioids, these results suggest that the prevalent negative feedback control of SG afferents activity is GABAergic, through both ionotropic and metabotropic receptors. In parallel, a serotonergic positive feedback could also modulate the type II neurons (Fig. 3d–f).

One of the ways by which the strength of activation of a synapse can be modulated is by regulating neurochemical delivery by pre-synaptic terminal through actions on exocytosis^27^. During this process, the cooperation between Synaptotagmins (*Syt*), complexins (*Cplx*), N-ethylmaleimide sensitive factor (*Nsf*), the SNAREs proteins synaptobrevin 1 (*Vamp1*), syntaxin (*Stx*), and SNAP-25 (*Snap25*), as well as Munc18-1 (*Stxbp1*) and Rab3a (*Rab3a*) is essential for synaptic targeting and membrane fusion^27^. Their enrichment within the type I neurons group supports a fast exocytosis at the auditory nerve endings (Fig. 3b, Supplementary Fig. 4c). In line with this, the enrichment of *Slc17a7* (VGLUT1) in type I afferents enables efficient recovery of synaptic vesicles during prolonged stimulation^28^ (Fig. 3b, Supplementary Fig. 4c). In addition, by regulating calcium availability, which triggers the fusion step^27^, the differential expression of calcium-binding proteins could also participate in the distinct firing dynamics of SG neurons^29^ (Fig. 3c).

Altogether, our data provide core molecular features of SG neuron types whose differential expression may underlie their input–output communication properties.

### Electrophysiological profiles of type I neurons

**Fig. 4:**
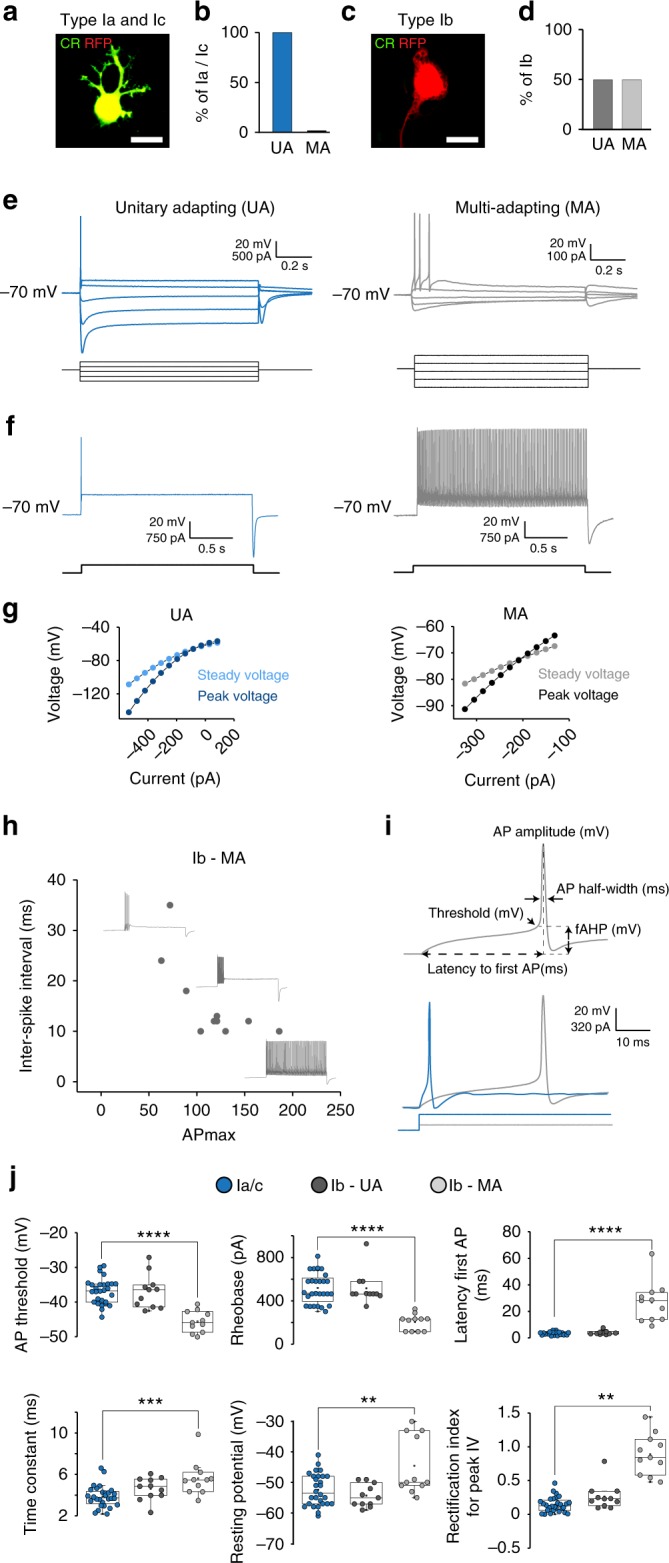
Electrophysiological characterization of SG neurons types. **a**, **c** Immunohistochemistry of RFP^+^ neurons after culture and patch-clamp recordings from SG neurons from P21, *PV*^*Cre*^;*R26*^*TOM*^ mice illustrating Ia/Ic types (CR^+^) and Ib type (CR^−^). **b**, **d** Correspondence of SG neuron types and their firing patterns, illustrating that all Ia/Ic neurons (*n* = 28) are unitary spike accommodating (UA) and 50% of Ib neurons (*n* = 11) are UA while the other 50% (*n* = 11) are multiple spikes accommodating (MA). **e** Representative whole-cell current-clamp recordings from UA and MA neurons from P21 SGNs. **f** Different accommodation rates and action potential firing patterns of representative UA (left) and MA (right) neurons in response to suprathreshold step current injections. **g** Graphs of the current–voltage relationship illustrating the state and peak IV responses for UA (left) and MA (right) types. Note steeper slope for peak voltage in MA cells than UA suggesting stronger rectification. **h** Plots of inter-spike interval (ISI) vs action potential (AP) max of the stained SG neurons, illustrating the diversity of type Ib neurons. **i** Schematic representation of measured action potential parameters (fAHP—fast after-hyperpolarization). Bottom, AP shape of UA (blue) and MA (gray) neurons showing different AP threshold and rheobase values, along with different AP kinetics (latency, duration, and fAHP). **j** Comparison of basic electrophysiological parameters highlighted in (**h**) between Ia/Ic, Ib UA, and Ib MA SG neurons. Data are represented as mean ± SEM (**P ≤ 0.01, *** *P* ≤ 0.001, **** P ≤ 0.0001; *t*-test between Ia/c UA and Ib MA population.) Scale bars: 20 μm (**a**,**c**)

We next asked whether the three distinct subclasses of type I SG neurons also exhibit unique electrophysiological properties. Whole-cell recordings were made from isolated SG neurons after dissociation to capture their key electrophysiological properties at the soma level. Analysis of current-clamp recordings revealed a high degree of diversity amongst the 133 recorded neurons in terms of action potential firing patterns and intrinsic passive and active properties. Two main groups could be easily distinguished based on their accommodation rate to step current injections: unitary spike accommodating/phasic cells (UA, *n* = 107 cells, 80% of neurons) and multiple spikes accommodating cells (MA, *n* = 26 cells, 20% of neurons, also characterized by a sustained firing over 200 ms long current steps) (Supplementary Fig. 5a), as shown at earlier stages^24^. UA cells were not able to fire more than a single action potential in response to a prolonged depolarizing step, while MA fired multiple action potentials with frequencies that increased by incrementing current intensity (Supplementary Fig. 5a). Post hoc immunostaining on 50 cells revealed that all Ia and Ic neurons (28 TOM^+^/CR^+^ neurons in a *PV*^*Cre*^;*R26*^*TOM*^ context) corresponded to UA type (Fig. 4a, b, e–g, Supplementary Fig. 5b), while the Ib population (22 TOM^+^/CR^−^ neurons) was equally comprising either UA (11 cells) or MA type (11 cells) (Fig. 4c, d, e–g) raising the possibility that the Ib type could be further subdivided. Moreover, neurons with an MA profile showed high variability in the number of spikes they fired with prolonged depolarization current (Fig. 4h).

To further describe the two SG neuron populations, we performed a more detailed analysis of their action potential properties. Only the Ib neurons with a MA profile differed significantly in most analyzed parameters (Fig. 4i–j, Supplementary Fig. 5c). In general, UA cells exhibited higher depolarization threshold compared to MA cells, their resting membrane potential was more hyperpolarized and they required stronger current injections to discharge action potentials. MA cells fired in response to smaller current injections, responded slowly and with a longer latency and had wider action potentials. Also, a slow after-hyperpolarization (AHP) was more prominent among MA cells. Other distinctive features of MA cells were their pronounced rectification of the current–voltage (*I*–*V*) relationship, larger Ih-mediated sag, shorter membrane time constant, and a high variability in their maximal frequency discharge, reaching up to 106 Hz (mean = 51 Hz).

To correlate the physiological data of SG neurons to their molecular profile, we found that *Kcnc2* (K_v_3.2) exhibited a contrasted expression profile in type Ib neurons, identifying two populations, which reinforces the possibility of a subdivision of type Ib in two subtypes (Supplementary Fig. 5d). One did not express *Kcnc2* while the other expressed variable levels of *Kcnc2*. K_v_3.2 has been shown to play an important role for sustaining repetitive firing^23,30^. Thus, these two populations could refer to the UA and MA types of Ib neurons, respectively. In line with this, type Ia and Ic neurons, which are UA type, did not express *Kcnc2* (Supplementary Fig. 5d). To the best of our analysis, *Kcnc2* was the only candidate gene in our neurotransmission-related gene data set to show this contrasted expression in type Ib neurons. In view of the importance of K_v_3 channels in the regulation of the firing properties of neurons^31^, it will be interesting to assess in vitro and in vivo the role of K_v_3.2 in the physiology of type Ib neurons and in hearing.

Although our data obtained on dissociated neurons do not reflect the actual biophysical properties of the post-synaptic SG neurons during synaptic transmission of inputs from IHCs, they do demonstrate a high heterogeneity of electrophysiological properties among SG neuron types. These neuron types could conceivably correspond to the auditory units with distinct thresholds to sound stimulation in vivo^5,8^.

### Spatial segregation of type I afferent terminals

**Fig. 5:**
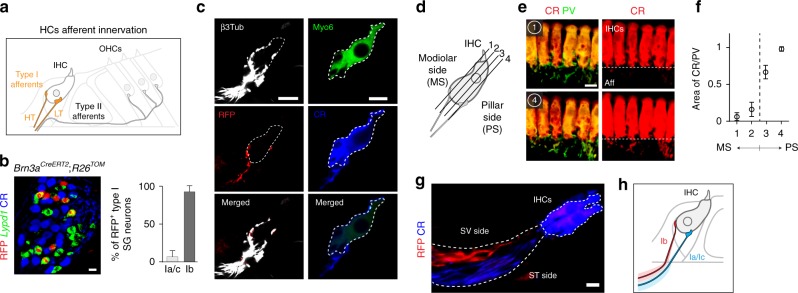
Innervation pattern of IHCs by type I neurons. **a** Sketch representing the afferent innervation of the mature organ of Corti, and illustrating the spatial segregation of the peripheral projections and synaptic contacts of high threshold (HT) and low threshold (LT) SG neuron fibers with IHCs. HT fibers innervate the modiolar side while LT fibers, the opposite, pillar side of IHCs. **b** Genetic labeling of Ib neurons using *Brn3a*^*CreERT2*^;*R26*^*TOM*^, injected with tamoxifen at P21 and analyzed on cross-section at P30. About 96% of RFP^+^ cells were *Lypd1*^+^ and were CR^−^, confirming their Ib identity (*n* *=* 3 animals). **c** In *Brn3a*^*CreERT2*^;*R26*^*TOM*^ mice, RFP^+^ Ib fibers innervate the modiolar side, while CR^+^ Ia and Ic fibers, the pillar side of IHCs. In the merged panel for the CR (Ia/Ic fibers) and Myo6 (IHC) staining, the IHC is shadowed to better visualize the innervation. **d** Schematic of the position of sections shown in **e** and **f**. **e** Whole mount staining of P21 cochlea, using CR and PV immunostaining in WT mice. The images show the presence of CR^+^ fibers on the pillar side (PS, section #1) and their absence on the modiolar side (MS, section #4) of IHCs, while PV^+^ afferents are observed on either side (Aff: afferents). **f** Quantification of the distribution of CR^+^ afferent fibers at different section levels of the IHCs (from the modiolar side to the pillar side) by measuring the area of the CR^+^ fibers within the area of PV^+^ fibers at different levels of the IHC innervation, as shown in **d** and **e** (*n* = 4 animals). Note that no CR^+^ fibers were observed outside the PV^+^ fibers area. **g** In *Brn3a*^*CreERT2*^;*R26*^*TOM*^ mice (see **b**), the peripheral projections of Ib (RFP^+^) and of Ia/Ic (CR^+^) neurons within the osseus lamina are segregated and occupy the scala vestibuli (SV) and scala tympani (ST) sides, respectively. **h** Schematic summary of the IHC innervation by type I afferents. Data are presented as mean ± SEM. Scale bars: 20 μm (**b**,**c**); 10 μm (**e**,**g**)

Multiple type I afferents (10–20 in mice) receive input from one IHC^32^—in comparison, a single type II neuron receives input from a dozen of outer HCs (OHCs)^1^. Remarkably, the position of these type I afferent terminals around the IHC circumference determines the fibers’ threshold sensitivity^8,33,34^. Fibers with high thresholds (HT afferents) to acoustic stimuli have been shown to contact the modiolar side (facing the modiolus) of the IHC while those with lower acoustic thresholds (LT afferents), their opposite, pillar side (facing the pillar cells)^8,9,34,35^ (see scheme in Fig. 5a). Differences in threshold sensitivity are important for expanding the dynamic range of the cochlea, and would provide a means for discriminating sounds in a background noise^5,34^. They also strongly suggest the existence of distinct ascending circuits and, consistent with this, specialized neuron types. To test whether the molecular differences we identified between SG neuron types could underlie the cellular basis of this functional diversity, we first made use of the high expression of *Pou4f1* (Brn3a) in type Ib neurons to selectively trace their nerve endings below IHCs using *Brn3a*^*CreERT2*^;*R26*^*TOM*^ mice and limiting tamoxifen exposure (a single injection at P21) (Fig. 5b). Indeed, a single injection of tamoxifen in P21 *Brn3a*^*CreERT2*^;*R26*^*TOM*^ mice lead to a sparse labeling of mostly type Ib neurons at P30 (94% of RFP^+^ type I neurons are Ib neurons, with 25% efficiency within the Ib population). Combined with CR immunostaining to label the projections of type Ia and Ic neurons, this sparse labeling strategy revealed on cross-sections and in cochlea whole mount that TOM^+^ Ib fibers consistently innervated the modiolar side of IHC, while CR^+^ Ia and Ic fibers, the pillar side (Fig. 5c, Supplementary Fig. 6a-c). Importantly, both CR and TOM positive fibers did not co-localize with synaptotagmin positive pre-synaptic nerve endings underneath the IHCs, excluding the possibility that these fibers were efferents (Supplementary Fig. 6b-c-e). A quantitative analysis of CR^+^ (only in Ia/Ic neurons) vs PV^+^ (in all neurons) fibers underneath the IHCs in cochlea whole mount of WT mice further confirmed the specific projection of Ib neurons to the modiolar and of the Ia/Ic neurons to the pillar (Ia/Ic) side of the IHCs (Fig. 5d–f, Supplementary Fig. 6d-e). Strikingly, the segregation of type I afferent projections was already defined in the osseus spiral lamina region, where peripheral axons of all SG neurons merge and project towards the sensory epithelium (Fig. 5g). Thus, Ib fibers were systematically positioned on the scala vestibuli (SV) side of the nerve bundle, while the Ia and Ic fibers, on the scala tympani (ST) side. Altogether, these data demonstrate that genetically distinct subclasses of type I SG neurons are associated with specific peripheral projection profiles with IHCs that correlate well with the distinction between LT and HT afferents^8,34^ (Fig. 5h).

### Neuronal diversity in the cochlea is established at birth

**Fig. 6:**
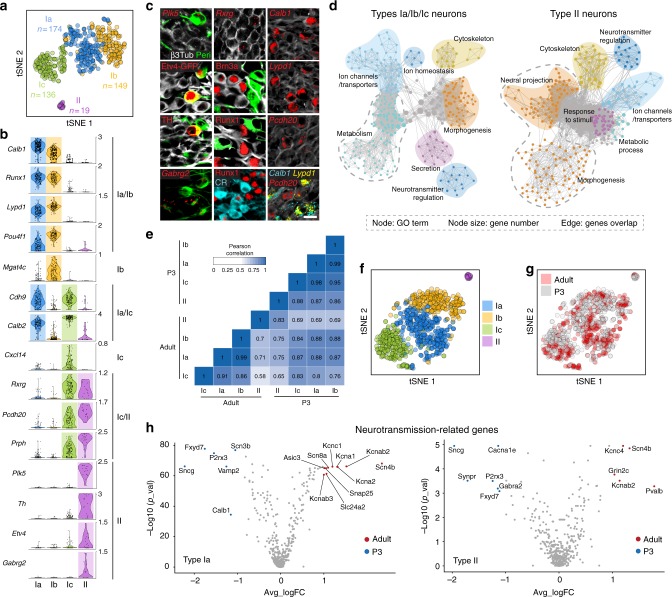
SG neuron types in new born mice and comparative analysis of their transcriptome with adult SG neurons. **a** t-SNE of SG neurons showing four different clusters at P3. **b** Violin plots showing the expression of marker genes in log-transformed scale. **c** In vivo validation of the identified neuron types by immunohistochemical and fluorescent in situ hybridization using identified marker genes in P3 cochlea. Type II SG neurons were identified by Peripherin (Peri), *Plk5*, *Etv4* (*Etv4*^*GFP*^ transgenic mouse), TH, and *Gabrg2*. Ia neurons were identified by *Pou4f1*, Runx1, and CR. Ib neurons were identified by Runx1 and *Pou4f1* and Ic neurons, by *Rxrg*, *Pcdh20*, and CR expression. *Lypd1* and *Calb1* expression could not distinguish type Ia from Ib neurons at this stage. Note that co-localization on sections could never be observed for markers expressed in different populations of neurons in the scNRAseq data. **d** Gene set enrichment analysis of P3 type I and type II SG neurons visualized by network. **e** Correlation analysis of SG neuron types from adult and P3 stages, using average expression of all differentially expressed genes as input. **f**, **g** Visualization of SG neuron types from adult and P3 stages using tSNE, revealing the conserved subclass identities between the two samples. **h** Volcano plots of gene expression differences between adult and P3 SG neuron types for type Ia (top panel) and type II (bottom panel). Genes differentially expressed in adult or P3 are marked by red or blue dots respectively. Scale bar: 20 μm (**c**)

In mice, pups are born deaf and become responsive to sound stimulation after P10^36^. Before the onset of hearing, neuronal activity in the SG is triggered by IHCs spontaneous activity^37^, which begins at about P4 and affects the maturation of the IHC-afferent synapses and certainly of different other cell types in the cochlea^37–39^. We therefore investigated the timing of establishment of SG neurons diversity, with respect to this critical maturation period. Unbiased clustering of 478 SG neurons from P3 cochlea mice revealed again four distinct types of SG neurons, in proportion similar (*P* > 0.05) to those observed in adult mice (Fig. 6a, Supplementary Fig. 7a-b). As in adult SG, the expression of transcription factors such as *Pou4f1* and *Runx1* was specific to the Ia and Ib types. The type Ic and II neurons were characterized respectively by a specific, transient expression of *Cxcl14* and *Etv4* (PEA3) (Fig. 6b, c; Supplementary Fig. 7c), distinguishing the two types from adult stages. Interestingly, both *Epha4* and *Prph*, described previously as early postnatal type I and type II neuron markers respectively^13,40^, were more widely expressed, with *EphA4* found in all neuron types, and *Prph*, in both type II and Ic neurons (Fig. 6b, Supplementary Fig. 7c-f; Supplementary Data 3, 4). Moreover, the four neuron types could be already identified at P0 (Supplementary Fig. 7g), suggesting that the molecular pathways that specify the generation of the four SG neuron types precede and are independent of the postnatal maturation of the organ of Corti and of spontaneous or stimulus-driven activity.

Through GSEA of P3 SG neuron types, we observed that “neurogenesis” and “morphogenesis/neuronal projection” were among GO terms with the highest representation in type II neurons (Fig. 6d). By contrast, the most enriched term in type I neurons concerned genes associated with metabolism, as was described earlier for the adult type I group (Fig. 6d). In line with this, a correlation analysis of adult and P3 SG neuron types revealed high similarity between the two stages (Fig. 6e–g). This was particularly visible for gene families related to neurotransmission (Fig. 6h, Supplementary Fig. 8), in which only a few number of genes showed different expression between P3 and adult stages. These results strengthen the idea that functional diversification of SG neuron types displayed prior to experience includes mature and distinct synaptic communication signatures that persist in adult neurons.

### Cell-to-cell communication machinery in P3 SG neurons

**Fig. 7:**
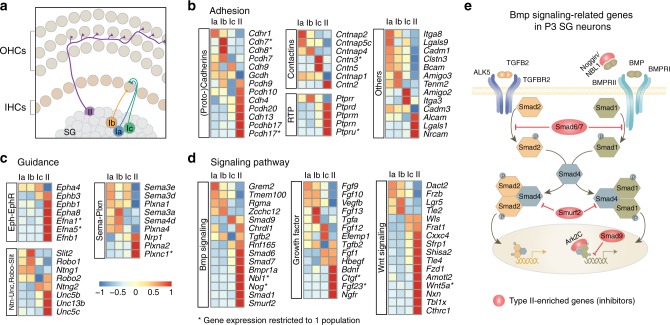
Functional signature of neonatal SG neuron types. **a** Schematic illustration of the mature connection pattern of auditory afferents with HCs. A single type II afferent travels through the OHCs area and receives synaptic inputs from several OHCs. A single-IHC makes synaptic contact with several type I neurons. Ia and one Ic afferent contact the pillar side, and Ib afferent contact the modiolar side of IHC. **b**–**d** Differential expression of adhesion-related genes (RTP, receptor tyrosine phosphatase), of guidance molecules and of genes linked to key signaling pathways including Bmp signaling, Wnt signaling, and growth factors among SG neurons at P3 (see also Supplementary Fig. 9). **e** Schematic illustration of the Bmp signaling using gene expression data from P3 SG neuron types. Note in red the type II enriched expression of genes coding for inhibitory proteins of the Bmp signaling

We next compared transcriptional differences amongst neonatal SG neuron types, focusing on gene sets commonly involved in neurodevelopmental processes, such as “axon guidance”, “adhesion”, and “signaling molecules”. These most likely participate to the establishment of their innervation pattern and to the specificity of their synaptic connections with HCs (Fig. 7a), efferents and central targets. As expected, the different types of SG neurons exhibited various combinations of gene expression (Fig. 7b–d, Supplementary Fig. 9). For instance, several genes of the Eph-Ephrin families (*Ephb1/3*, *Epha8*, *Efna1/5*, *Efnb1*) were particularly enriched in type II neurons, likely participating in the intricate growth of their projections within the OHCs area (Fig. 7a, b). Interestingly, several members of the cadherin, proto-cadherin and contactin cell surface molecules, which are critical in the control of synaptic partner specificity, displayed very distinct expression profiles across the four populations of neurons^41^ (Fig. 7b). In the cadherin family for instance, *Cdh7* and *8* were enriched in type Ib, *Cdh9* in types Ia and Ic and *Cdh4* and *13*, in type II neurons. For those categories of molecules, their relative levels of expression in different neighboring cells can generate discrete, local synaptic connection patterns. Thus, their interactions between synaptic partners could participate in the correct matching of each afferent types with particular cellular domains of HCs and efferent endings during development^42–44^.

We also found that whereas all SG neurons expressed the machinery necessary for the activation of the TGFB signaling, many inhibitory modulators of this pathway (*Smad6*, *Smad7*, *Nog*, *Nbl1*, *Smad9*, *Smurf2*) were particularly enriched in the type II neurons, arguing for a specific role of this signaling only in type I neurons (Fig. 7d, e). More generally, each of the major signaling pathways manifested differential expression amongst SG neuron types, highlighting their potential contribution to the characteristic connection patterns and physiological properties of each cell type.

Altogether, our results provide an extensive set of differentially expressed genes among neonatal SG neurons that will help future investigations of the molecular programs that control the diversification and connection patterns of SG neuron types.

## Discussion

Despite evidence supporting physiological diversity of primary auditory neurons, their molecular identification and thus the study of their individual function in the neuronal encoding of the distinct properties of sounds has remained unresolved. Using unbiased single-cell RNA profiling, morphological, and physiological analyses, we provide evidence for molecularly distinct types of auditory neurons in the cochlea as well as an extensive resource for future studies in hearing research.

Our results identified four distinct types of adult SG neurons, including three novel subclasses of type I neurons (Ia, Ib, and Ic neurons) and the type II neurons, which can be distinguished based on unique and combinatorial molecular profiles. These four types of SG neurons exist at birth, which strongly suggests that initial neuronal diversification in the cochlea is established by molecular processes independent of neuronal activity or sensory experience.

We found that a comprehensive analysis of the combinatorial expression of genes involved in neuronal transmission reveals a specific molecular profile of each neuron type that underlies their unique transcriptional signature of input/output communication. These particular profiles agree with well-studied physiological properties of SG neurons, such as their metabolic status, preference for AMPA-mediated or kainate-mediated synaptic transmission or their expression of various VGICs or receptors to efferent-derived neurotransmitters and neuromodulators^2,16,18,25^. They further extend the molecular characterization of SG neurons but also, more important, they provide an extensive cell to cell phenotype that can be used to predict or confirm physiological features.

For instance, the transcriptome of type II neurons suggests that they would be part of a particular response to a local stress (Fig. 2b), which is consistent with their activation by ATP and closure of K_v_7 potassium channels induced by damage of the organ of Corti^10,11^. Interestingly, our data show that amongst K_v_7 channels and purinergic receptors (P1, P2X, and P2Y receptors), only *Kcnq2* (K_v_7.2) and *P2rx4* (P2X4) and to a lesser extent, *P2rx7* (P2X7), which both code for ATP-gated ion channels (P2X receptors), are expressed in adult type II neurons (Supplementary Data 1). On the other hand, P2Y receptors, which are metabotropic and can be activated by ATP or UTP, have been shown to be upregulated in all SG neurons following noise trauma^45^. P2Y receptors activation can regulate the excitability of neurons via the inhibition of the M-current, which is mediated by members of the K_v_7 channels and reduces neuronal excitability^46–49^. Thus, this suggests a two-step activation mechanism of type II neurons following noxious noise exposure: a first through glutamate (via AMPA/Kainate receptors) and ATP (via P2X receptors) released from OHCs and the damaged organ of Corti, respectively, and a second (likely delayed) by inactivating K_v_7.2-mediated M-current and thus sensitizing type II neurons through a specific UTP–P2Y metabotropic pathway. Interestingly, we also discovered that while serotonin is expressed in both the lateral and medial efferent systems^50^, only the type II neurons express receptors for serotonin (*Htr2c* and *7*, coupled to G_q_/G_11_ and G_s_, respectively), suggesting a third mode of type II neurons excitation or sensitization by serotonin receptor activation (Supplementary Fig. 10).

The diversity of type I SG neurons has been described for decades, with cells showing distinct connection pattern and firing properties^5,8,9,24,33,34,51^. So far however, the cellular and molecular mechanisms proposed to underlie these differences involved variation in ribbon size at the IHC-afferent synapse, pre-synaptic specialization at the IHC active zone or the patterns of lateral efferents innervating the post-synaptic bouton of auditory afferents^34,52,53^. Although these mechanisms could exert significant roles on the sensitivity differences among auditory afferents, our data demonstrate the existence of three genetically distinct subclasses of type I neurons that could reflect specific coding of the vast range of intensity levels the auditory system processes^5^. Their molecular profile, connection and electrophysiological patterns strongly suggest that the Ia and Ic populations represent the LT auditory afferents while the Ib type, the HT afferents. Several observations support this hypothesis. First, peripheral projections of Ib and of both the Ia and Ic afferents travel separately to innervate the modiolar and the pillar sides of IHCs, respectively, a segregation previously associated with the different threshold sensitivities of the auditory afferents^1,2,8,33,34^. Second, the Ib neurons display a wide range of accommodation and spiking rates, which has been previously described and could provide a cellular substrate to encoding various stimulus intensities^24^. Third, *Kcnc2* (K_v_3.2), whose expression and function characterize neurons capable of sustained or repetitive firing^23,30^, is uniquely and variably expressed in the Ib population and might thus contribute to the molecular setting that controls the various firing patterns of Ib neurons. Finally, the proportion of Ib neurons increases in the cochlea of the rat (Supplementary Fig. 11) which, as the cat and macaque monkey, shows finer sensitivity in detecting intensity changes compared to mouse^54^. This could suggest that the more HT SG neurons, the finer sensitivity to intensity changes. Together, our results are thus consistent with the general idea of distinct specialized populations of SG neurons that would provide a neuronal substrate to process sound intensity information^5,8,33,34,51^. Moreover, they suggest that, similarly to what has been shown in the visual system^55^, within a particular neuronal population (i.e., Ib neurons), the continuum of variable biophysical properties of neurons could in fact be genetically determined so that specific groups of few neurons would be tuned to a particular range of intensities. This also supports future work aimed at validating in vivo the physiological role of Ib neurons in sound intensity coding and at resolving whether the Ia and Ic populations represent two functionally distinct populations or reflect certain local environment.

Overt hearing loss and self-perceived hearing handicap, which includes speech-in-noise recognition disabilities, tinnitus, and hyperacusis are often associated with degeneration or dysfunction of auditory afferents. Moreover, recent work showed that HT SG neurons are more vulnerable to loud sound^56,57^, and suggested that a progressive HT neuropathy might represent an important contributor to the hearing handicap observed with ageing^58^, highlighting a selective vulnerability of HT SG neurons. Our comprehensive transcriptomic characterization of the four types of SG neurons will thus facilitate comparative analysis of homologous cell types with other species, but also linking gene expression to cellular function and auditory perception as well as their alteration with hearing disorders. In this context, future studies will be needed to also identify the dedicated output neurons and associated ascending circuits needed to process centrally the peripheral information encoded by each individual SG neuron type.

## Methods

### Mouse strains

Wild-type C57BL/6 mice were used unless specified otherwise. *Rosa26*^*TOM*^ (Ai14, Stock No: 007914, C57Bl/6 background), *PV*^*Cre*^ (Stock No: 017320, C57Bl/6 background), mice were ordered from The Jackson Laboratory. *EphA4*^*GFP*^ (Stock No: 011107-UCD, mixed Swiss Webster and C57bl/6 background) was ordered from MMRRC. *Brn3a*^*CreERT2*^ (mixed 129Sv and C57BL/6 background, from CCTEC, Cornell) mice was described elsewhere^59^. Animals were group-housed, with food and water ad libitum, under 12-h light–dark cycle conditions. All animal work was performed in accordance with the national guidelines and approved by the local ethics committee of Stockholm, Stockholms Norra djurförsöksetiska nämnd. Animals were killed when reaching a score of 0.3 on Karolinska Institute’s extended health assessment list. Animals used for this study were of either sex for postnatal experiments, and males from P17 onwards.

### Tamoxifen-induced sparse labeling

Tamoxifen induction of *Brn3a*^*CreERT2*^;*Rosa26*^*tdTOM*^ mice was performed by single intraperitoneal injection of 2 mg per 25 g.bw into P21 mice. Mice were harvested at P30, and efficiency of recombination was analyzed using immunostaining for RFP and calretinin (Ia and Ic neurons) and in situ hybridization for *Lypd1* (Ib neurons) on spiral ganglion sections. The degree of recombination in spiral ganglion neurons was very low and variable between animals and litters. Only animals showing a recombination efficiency of about 25% within the Ib population of cochlear neurons were considered for analysis. In these animals, ~94% of RFP^+^ recombined type I neurons were Ib neurons, confirming the specificity of our sparse labeling strategy for the Ib afferents.

### Single-cell isolation

Spiral ganglia were dissected and collected in Leibovit’z L-15 medium (Life technologies) on ice. Then the spiral ganglia (SG) were incubated in papain–DNAse solution (1.5 ml of papain at 1 mg ml^−1^, 0.5 ml of DNAse at 0.1%) for 35 min (for P3 samples) or 25 min (for Adult samples) at 37 °C, shaking at 700 RPM. After spinning down the samples at 400 RCF for 10 min, the supernatant was removed and replaced by Dulbecco’s modified Eagle’s medium (DMEM) F-12 (Life technologies). SG were physically triturated using three different sizes of pipettes previously coated with 0.2% bovine serum albumin until the solution homogenized. The cell suspension was then filtered through 70 μm cell strainer (BD Biosciences) to remove clusters of cells.

### Fluorescence-activated cell sorting

Single Tomato or GFP positive cells were sorted by FAC sorting into individual wells of 384-well plates containing lysis buffer. Plates containing single cells were frozen immediately on dry ice and stored at −80 °C. We used six animals (12 cochleae) per experiment.

### Cell culture

After centrifugation of the single cells, pellets were reconstituted in 1 ml of culture media [Neurobasal-A, supplemented with 2% B27 (v/v), l-glutamine 0.5 mM, penicillin 100 U ml^−1^; Invitrogen] complemented with 10 ng µl^−1^ of BDNF and NT3 (Sigma-Aldrich) and 10% of fetal bovine serum. Cells were plated on square glass coverslips previously coated with poly-d-lysine and laminin (Nordic Biosite). SG neurons were cultured for 48 h to allow the detachment of Schwann cells from neuronal cells and the culture media were changed every 24 h.

### Single-cell RNA sequencing

Smart-Seq2 protocol was performed on single isolated cells by Eukaryotic Single Cell Genomics Facility at SciLifeLab, Stockholm. The protocol was described previously^60,61^. A total of 487 adult SG neurons were isolated from *PV*^*cre*^,*R26*^*TOM*^ mice and for P3 stage, a total of 478 SG neurons were isolated: 359 from *PV*^*cre*^,*R26*^*TOM*^ mice (P3, sample A), and 119 SG neurons, from *EphA4*^*GFP*^ mice (where GFP expression is labelling all SG neuron types without distinction at P3, see Supplementary Fig. 7) (P3, sample B). RNAseq data from sample A and B were indistinguishable and were therefore merged to increase the sample size.

### Electrophysiological recordings

Recordings were made from neuronal somata at room temperature. Neurons were obtained from P21 to P25 *PV*^*Cre*^;*R26*^*TOM*^ mice. The bath solution was composed of (in mM): 125 NaCl, 25 glucose, 25 NaHCO_3_, 2.5 KCl, 2 CaCl_2_, 1.25 NaH_2_PO_4_, 1 MgCl_2_ and saturated with 95% oxygen and 5% CO_2_. Cells were visualized using IR-DIC microscope (Olympus, TYO, Japan) and digital camera. Tomato-expressing PV^+^ cells were identified by switching from infrared to epi-fluorescence mode using a LED source and LED control module (Mightex BioLED, CA, USA). Patch pipettes with resistances of 6–10 MΩ were pulled with Flamming/Brown micropipette puller P-1000 (Sutter Instruments Co, Novato, CA) and filled with intracellular solution containing (in mM): 130 K-gluconate, 5 KCl, 10 HEPES, 10 Na_2_-Phosphocreatine, 4 ATP-Mg, 0.3 GTP-Na and with pH adjusted with KOH to 7.3 and osmolarity: 288 mOsm l^−1^. Liquid junction potential was not corrected for. Recordings were performed with pipette capacitance and access resistance compensated throughout the experiment. Acceptable current-clamp recordings had to meet the following criteria: low noise level, stable membrane potential, and overshooting APs (magnitudes of ≥60 mV from baseline) and access resistance below 20 MΩ. If any of these parameters changed during an experiment, the remaining data were not further analyzed. Recordings were amplified using multiclamp 700B amplifiers (Molecular Devices, CA, USA), filtered at 2 KHz, digitized (10–20 KHz) using ITC-18 (HEKA Elektronik, Instrutech, NY, USA), and acquired and analyzed with Igor Pro (Wavemetrics, OR, USA). Remote control SM7 system (Luigs Neumann, Germany) was used to control manipulator, read, and save *XYZ* coordinates of recorder cells in the dish for later identification.

### Stimulation protocols

The intrinsic electrical properties were determined by a series of hyperpolarizing and depolarizing somatic current injection protocols, during whole-cell patch-clamp recordings, designed to capture their key active and passive electrical properties. All recordings were carried out in current-clamp mode and because dependency of the accommodation rate on the holding membrane potential was not observed at this post hearing stage, all cells were recorded at −70 mV. Several parameters were obtained from recorded cells**:** resting membrane potential was measured at the onset of whole-cell recording. IV (the current–voltage relationship) was extracted by a series of subthreshold current injections with addition scaling factor multiplying steps accordingly to resistance and rheobase to obtain optimal resolution for each cell*. Input resistance* for steady state was calculated as the slope of the regression line fit to steady-state membrane potential responses to −5 pA current injection from rest. Voltage threshold for evoking action potential (AP) was measured as the value of the membrane potential at which its first derivative (d*V*/d*t*) crossed 10 mV s^−1^ in response to step depolarization from resting potential and rheobase was the minimal depolarizing current to reach threshold and elicit an action potential. Membrane time constant was determined by fitting the decay phases of hyperpolarizing pulses to an exponential function. AP max: maximal number of AP elicited in response to 200 ms lasting suprathreshold step current reaching three times stronger current than rheobase. Inter-spike interval (ISI): measured as the difference in time between the onsets of the first two APs. Parameters assessed included also action potential waveform AP duration: average time for the first AP from onset to the same voltage during offset. AP onset: defined as the time near the voltage inflection when the voltage changes more than 0.5 mV within 50 µs. AP latency: time from the threshold to peak of the AP. AP duration half-width: average time for the first AP from half amplitude to the same voltage during offset. Sag, the response to a hyperpolarizing current step: was measured as the difference between exponentially extrapolated voltage and steady-state voltage and analyzed to reveal the existence of hyperpolarization-dependent inward currents. Slow after-hyperpolarisation (s-AHP): measured following rapid discharge. Fast AHP: amplitude from first AP onset to minimum voltage. Rectification index for steady state (or peak) IV: change in input resistance at steady-state (or peak) voltage.

### Immunohistochemistry and in situ hybridization

All data are from P21 mice, unless specified. Cochleae were fixed in 4% paraformaldehyde, O/N (for in situ hybridization) or 2 h (for immunohistochemistry) at 4 °C. After fixation, cochleae older than P10 were incubated in decalcification cocktail (fresh 5% EDTA, 0.4% paraformaldehyde in PBS) O/N at 4 °C. Cochleae were then incubated in 30% sucrose O/N at 4 °C. Finally, half of the sucrose solution was replaced by optimal cutting temperature (OCT) compound for ON, rolling at 4 °C. Specimens were then mounted in OCT and sectioned at 14 µm. RNA in situ hybridization experiments were performed using RNAscope®, an RNA in situ hybridization technique described previously^62^. Paired double-Z oligonucleotide probes were designed against target RNA using custom software. The RNAscope® Reagent Kit (Advanced Cell Diagnostics, Newark, CA) was used according to the manufacturer’s instructions (kit version 1). Frozen fixed tissue sections were prepared according to manufacturer’s recommendations. Each sample was quality controlled for RNA integrity with a probe specific to the housekeeping gene *Ppib*. Negative control background staining was evaluated using a probe specific to the bacterial *DapB* gene. The probes used in this study are summarized in Supplementary Table 2. For immunohistochemistry, sections were permeabilized, blocked and incubated O/N at 4 °C with 0.5% Triton X-100, 10% normal donkey serum (Fischer Scientific) and the appropriate concentration of primary antibodies in PBS, followed by three rinses in PBS and labeling with AlexaFluor-conjugated secondary antibodies (1:500, Invitrogen) in 0.5% Triton X-100, 10% normal donkey serum in PBS for 2 h at room temperature. Sections were then washed three times in PBS and mounted with fluorescent mounting medium (Dako). For Runx1 staining, cryostat sections were permeabilized with 1.5% Triton X-100 for 2 h, followed by three washes with PBS and incubated with primary antibody in 0.3% Triton X-100/3% normal serum in PBS for 72 h at 4 °C, three washes in PBS and incubated with primary antibody in 0.3% Triton X-100/3% normal serum in PBS for 3 h at room temperature and then overnight at 4 °C.

### Quantification of the neuronal fibers

Whole mount cochlea were stained by using calretinin (CR) to target Ia/c fibers and parvalbumin (PV) for all type I fibers underneath the IHCs. For each cochlea, three areas with seven IHCs per area from mid-basal levels were imaged (Z-stacks through the organ of Corti) and analyzed. The organ of Corti was 3D reconstructed, and the baso-apical orientation of the IHCs was assessed by using the stereocilia of the apical region of the IHCs as landmarks. The IHC side facing the OHCs was considered as the pillar side, and the IHC side facing the SG neurons, the modiolar side. Each Z-stack section was analyzed using ImageJ, first by defining the area of interest (below the IHCs). Automated selection of the immunostained area for either PV or CR positive fibers was done using “analyze particle” in ImageJ and measured.

All sections were imaged using a Zeiss LSM 700 confocal microscope. Antibodies used, together with their concentration, source and catalog number are summarized in Supplementary Table 1.

### Statistical analysis of imaging data

Statistical analysis was performed using GraphPad Prism. For neuronal counting and soma area quantifications in cochlea sections, we used ImageJ software. ABout 6–8 randomly selected sections per animal (for basal, medial and apical region) from at least three different animals were used for quantification. The results are shown as mean ± SEM. as indicated in figure legends. The statistical test performed is reported in the figure legend. *t*-tests were two-sided. Legend for significance: *P ≤ 0.05, **P ≤ 0.01, ***P ≤ 0.001. No animals or data points were excluded from the analyses. No statistical methods were used to pre-determine sample size but our sample size are similar to those generally employed in the field. Randomization was used whenever possible.

### Single-cell RNAseq data analysis

P3 and adult data were analyzed with the same pipeline, though with minor differences in the parameter settings. Here we describe the analysis of adult data specifically. The raw data of single-cell RNA sequencing were processed with standard pipeline by Eukaryotic Single Cell Genomics Facility at SciLifeLab, Stockholm. Most of the downstream analysis was done using R software package Seurat v2.0 (https://github.com/satijalab/seurat; http://satijalab.org/seurat/).

Considering the distribution of genes identified in every cell, we selected cells with at least 5000 (P17 and P21 data) or 6000 (P33 data) unique genes detected for downstream analysis. This step removed empty wells and low-quality cells, leaving 316, 137, 34 cells for P17, P21, and P33 data, respectively. Genes expressed in less than three cells were also removed from analysis. To be able to integrate different data sets, we used a global normalization strategy incorporated in SEURAT package to make the data comparable between different cells and between different SMART-seq2 runs. The gene expression measurements within each cell were scaled by a constant factor 10,000, then natural-log transformed. This generated a new gene expression matrix $y_{i,j}{\mathrm{ = log}}(\frac{{x_{i,j}}}{{Z_j}} \ast 10,000)$, where *x*_*i,j*_ is the count of gene i in cell j and *Z*_*i,j*_ is the total counts of all genes in cell j.

We calculated the dispersion (ratio of variation to mean) of genes and selected those highly variable genes from each data set. The variable genes were used for alignment of different data sets. We also regressed out the cell–cell variation in gene expression driven by “percent.mito (the percentage of mitochondrial gene content)” using ScaleData function.

To reveal the shared sources of biological variation in order to identify common subpopulations across different stages, we used the integration tool for single-cell RNAseq data sets provided by Seurat package version2.0. This tool has shown better performance over other benchmarking alignment and batch correction tools, e.g., limma, ComBat^63^. It comprises Canonical Correlation Analysis (CCA) which is a method of inferring relationship from two matrices. Given two groups of cells X = (*X*_1_, *X*_2_,…, *X*_*m*_) and Y = (*Y*_1_, *Y*_2_,…, *Y*_*n*_), and if there were correlations among them, the analysis would determine a set of canonical variates (CVs, referred to as CCs in Seurat package), storing linear combinations of *X*_*i*_ and *Y*_*j*_ which have maximal correlation with each other. Then CCs could be used for downstream analysis, including *t*-distributed stochastic neighbor embedding (*t*-SNE) and subtype clustering. We started with aligning P17 and P21 data together. Using a union of most variable genes from both data sets as the input for CCA, we selected the first ten CCs to approximate all CCs which project both data sets into the maximally correlated subspace. Then we added P33 data into the union of P17 and P21 data, repeated the CCA and selected the first nine CCs to align the CCA subspaces. As such, all cells from different stages were integrated together.

In order to obtain an overview of heterogeneity in the integrated adult data, we ran function RunTSNE using the first 9 CCs to visualize cells in the two-dimensional space and used function FindClusters (resolution = 1) to identify clusters of cells.

To identify differentially expressed genes in each cluster, we used likelihood-ratio test^64^ implemented in function FindMarkers. The parameters “min.pct = 0.1” and “thresh.use = 0.1” are chosen to select genes expressed in more than 10% of cells and with average expression level over 0.1 (normalized data) in the given cluster. The genes were ranked by *P*-value from low to high, with lower *P*-value indicating more differentially expressed genes.

### Gene Set Enrichment Analysis visualized by network

Top 500 of type I SG neurons or type II SG neurons specific genes were used as input for GSEA using Cytoscape^65^ and its plugin Bingo^66^. The most up-to-date gene ontology file “go-basic.obo” and gene association file “gene_association.mgi” were downloaded directly from Gene Ontology Consortium website instead of using Bingo’s default ontologies or associations. The following evidence codes were discarded from analysis: IEA (inferred from electronic annotation), ISS (inferred from sequence similarity).

Significant GO terms (*P*-value < 0.05, corrected using Benjamini & Hochberg False Discovery Rate (FDR) correction) were used as input for Cytoscape’s plugin EnrichmentMap to generate a network where mutually overlapping gene sets cluster together. Following parameters were used: *P*-value cutoff 0.001, FDR *Q*-value cutoff 0.05, similarity cutoff with Jaccard coefficient 0.25.

Genes which were differentially expressed in at least one but not all SG neuron types were pooled together (Supplementary files). We retrieved the genes annotated with specific GO terms (Supplementary Table 3) and showed expression of them in different SG neuron types by heat map.

### Comparison between P3 and adult

The average gene expression of SG neuron types at P3 and adult were used to compute Pearson’s correlation coefficient. Differentially expressed genes between P3 and adult were identified using Wilcoxon rank sum test. *P*-value and log fold change of genes were used to compute the volcano plots. We have compared the volcano plots obtained using the *P*-values with those using the multiplicity adjusted *P*-values and found no difference in the results (genes differentially expressed between P3 and adult in the neurotransmission-related category of genes). The *P*-value for all volcano plots are shown in the Fig. 6 to keep the consistency between all neuronal subclasses, since the type II subclass has only seven cells in our data set, and multiplicity adjusted *P*-values cannot be applied for this group.

## Electronic supplementary material

Supplementary Information

Peer Review File

Supplementary Data 1

Supplementary Data 2

Supplementary Data 3

Supplementary Data 4

Supplementary Data 5

## Data Availability

The accession number for the data used in this article is GEO: GSE117055 . Expression profile of any genes among SG neuron types can be visualized at: http://ki.se/en/neuro/lallemend-laboratory. Data that support the findings of this study are also available from the corresponding author upon request. The authors declare that all data supporting the findings of this study are available within the manuscript or its Supplementary Files or are available from the corresponding author upon reasonable request.
