# Supplementary material for: Neuronal heterogeneity and stereotyped connectivity in the auditory afferent system
Source: Nat Commun. 2018 Sep 12;9:3691. doi: 10.1038/s41467-018-06033-3 (PMC6135759; doi:10.1038/s41467-018-06033-3)
Supplement: Supplementary file 1 — Supplementary Information [file 41467_2018_6033_MOESM1_ESM.pdf]

Description of Additional Supplementary Files

File Name: Supplementary Data 1
Description: Adult genetic markers expression profile of SG neurons.

File Name: Supplementary Data 2
Description: Adult markers of SG neurons subtypes.

File Name: Supplementary Data 3
Description: P3 genetic markers expression profile of SG neurons.

File Name: Supplementary Data 4
Description: P3 markers of SG neurons subtypes.
