# Supplementary material for: Neuronal heterogeneity and stereotyped connectivity in the auditory afferent system
Source: Nat Commun. 2018 Sep 12;9:3691. doi: 10.1038/s41467-018-06033-3 (PMC6135759; doi:10.1038/s41467-018-06033-3)
Supplement: Supplementary file 3 — Supplementary Data 1 [file 41467_2018_6033_MOESM3_ESM.pdf]

Supplementary Information

‘Neuronal heterogeneity and stereotyped connectivity in the auditory afferent system’

Charles Petitpré, Haohao Wu, Anil Sharma, Anna Tokarska, Paula Fontanet, Yiqiao Wang, Françoise Helmbacher, Kevin Yackle, Gilad Silberberg, Saida Hadjab & François Lallemend

Supplementary Figures (1 – 11)

Supplementary Tables (5 – 7)

Supplementary References

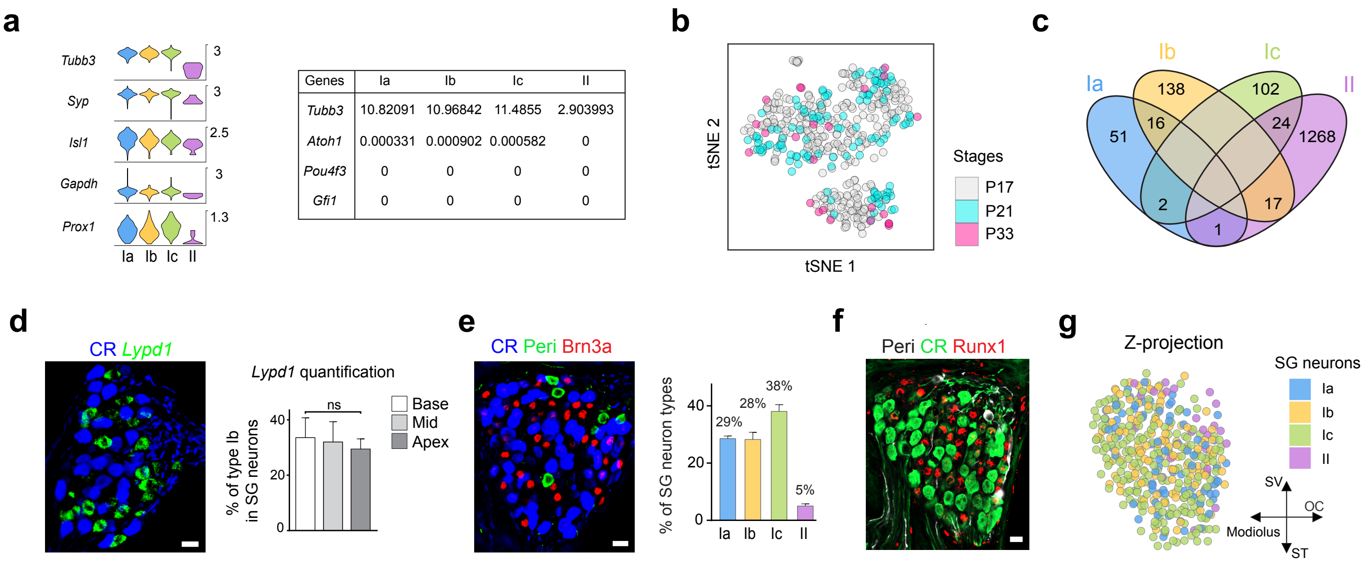

Genes	Ia	Ib	Ic	II
Tubb3	10.82091	10.96842	11.4855	2.903993
Atoh1	0.000331	0.000902	0.000582	0
Pou4f3	0	0	0	0
Gfi1	0	0	0	0

Supplementary Figure 1. Transcriptomic analysis of adult SG neurons. (**a**) Violin plots depicting expression of specific control genes in our 4 populations of SG neurons in log-transformed scale. Note enrichment of *Tubb3* (β3-tubulin) and *Prox1* in the type I compared to type II SG neurons. Importantly, as shown in the table, in comparison to the control neuronal marker *Tubb3*, hair cells specific markers such as *Pou4f3*, *Atoh1* and *Gfi1*[1,2,3] were not found to be expressed in our scRNAseq, confirming the neuronal identity of our cells. (**b**) Visualization of SG neurons from different stages (P17, P21 and P33) using tSNE, revealing the conserved subclass identities between stages. (**c**) Venn diagram of the differentially expressed genes of the 4 different SG neuron populations. (**d**) Immunohistochemistry for calretinin (CR, marking Ia/Ic neurons) and ISH (RNAscope) for *Lypd1* (marking Ib neurons) on P21 SG sections revealing the mutually exclusive expression of the 2 markers. Graph shows the quantification of Lypd1^{+} Ib neurons (in % of β3-tubulin^{+} neurons, all neurons), at basal, middle and apical regions of the cochlea (P = 0.1884, unpaired *t* test, n=3; data are presented as mean ± SEM). (**e**) Immunohistochemistry for peripherin (Peri), Brn3a and CR on P21 SG sections: Ia neurons, CR^{+}/Brn3a^{+}; Ib neurons, CR^{-}/Brn3a^{+}; Ic neurons, CR^{+}/Brn3a^{-}; II neurons, Peri^{+}. Graph shows the proportion of each SG neuron subclass in basal cochlear turn (n=4, data are presented as mean ± SEM). (**f**) Representative immunostaining for peripherin, CR and Runx1 on P21 SG section, illustrating the spatial patterning of the different neuronal subclasses within the SG. (**g**) Schematic representation of the projection of 6 images from P21 SG sections (from basal cochlea region) immunostained as in (**f**); dots indicate SG neurons and color codes reveal neuron subclass and illustrate the spatial pattern of the four subclasses of SG neurons. SV, scala vestibule; ST, scala tympani; OC, organ of Corti. Scale bars: 20 μm (**d**,**e**,**f**).

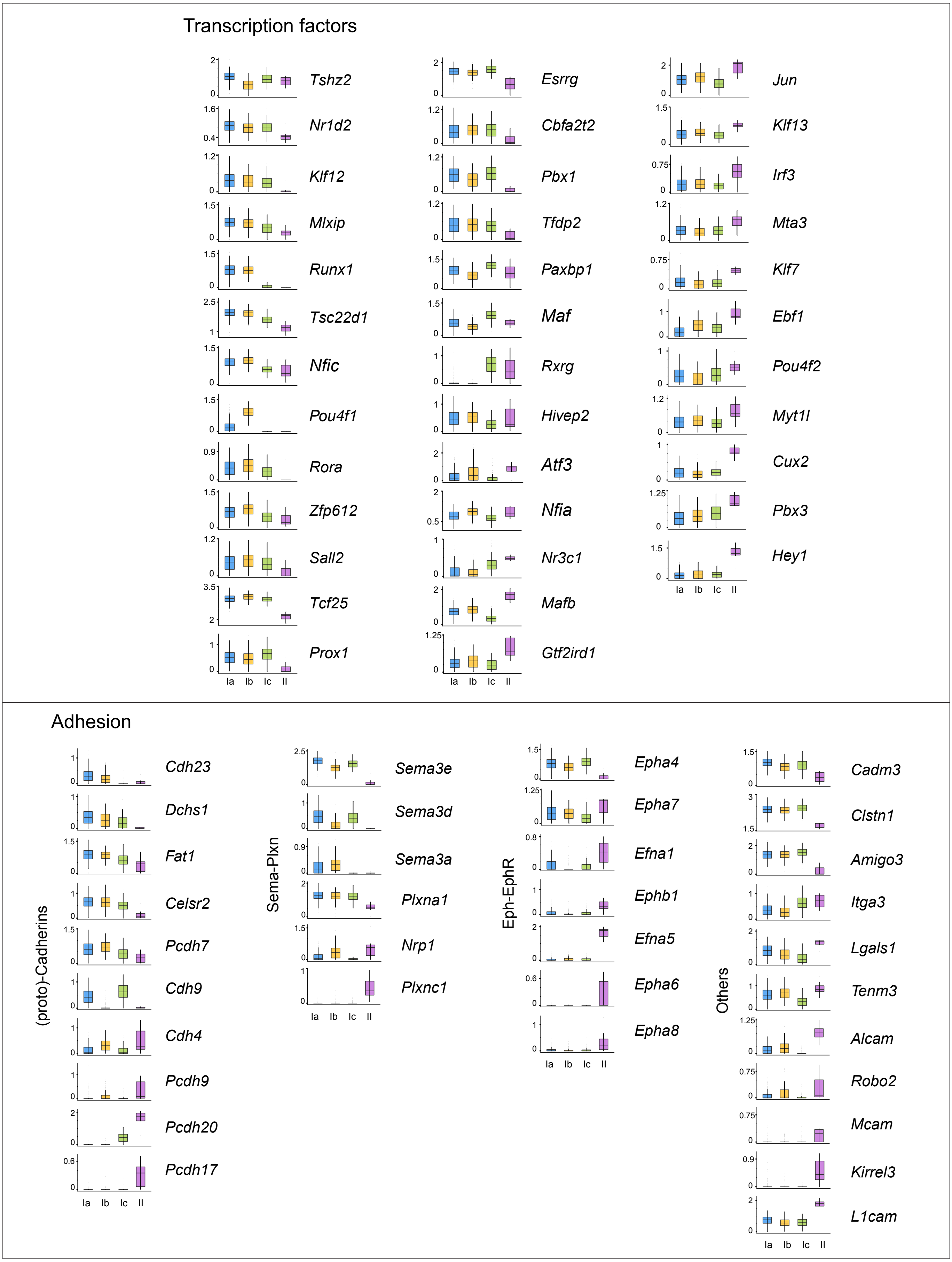

Supplementary Figure 2. Differential expression of Transcription factors and of cell adhesion related genes in adult SG neurons. Boxplots representing gene expression of transcription factors genes and genes related to adhesion such as Cadherins, Semaphorins and Ephrins families. The lower and upper hinges represent the first and third quantiles respectively, so the box spans the inter-quartile range (IQR). The horizontal line inside box corresponds to median. The upper whiskers extend to the largest value no further than 1.5*IQR from the upper hinge, while the lower whiskers extend to the smallest value at most 1.5*IQR from the lower hinge. Dots represent the “outliers” beyond the range of whiskers.

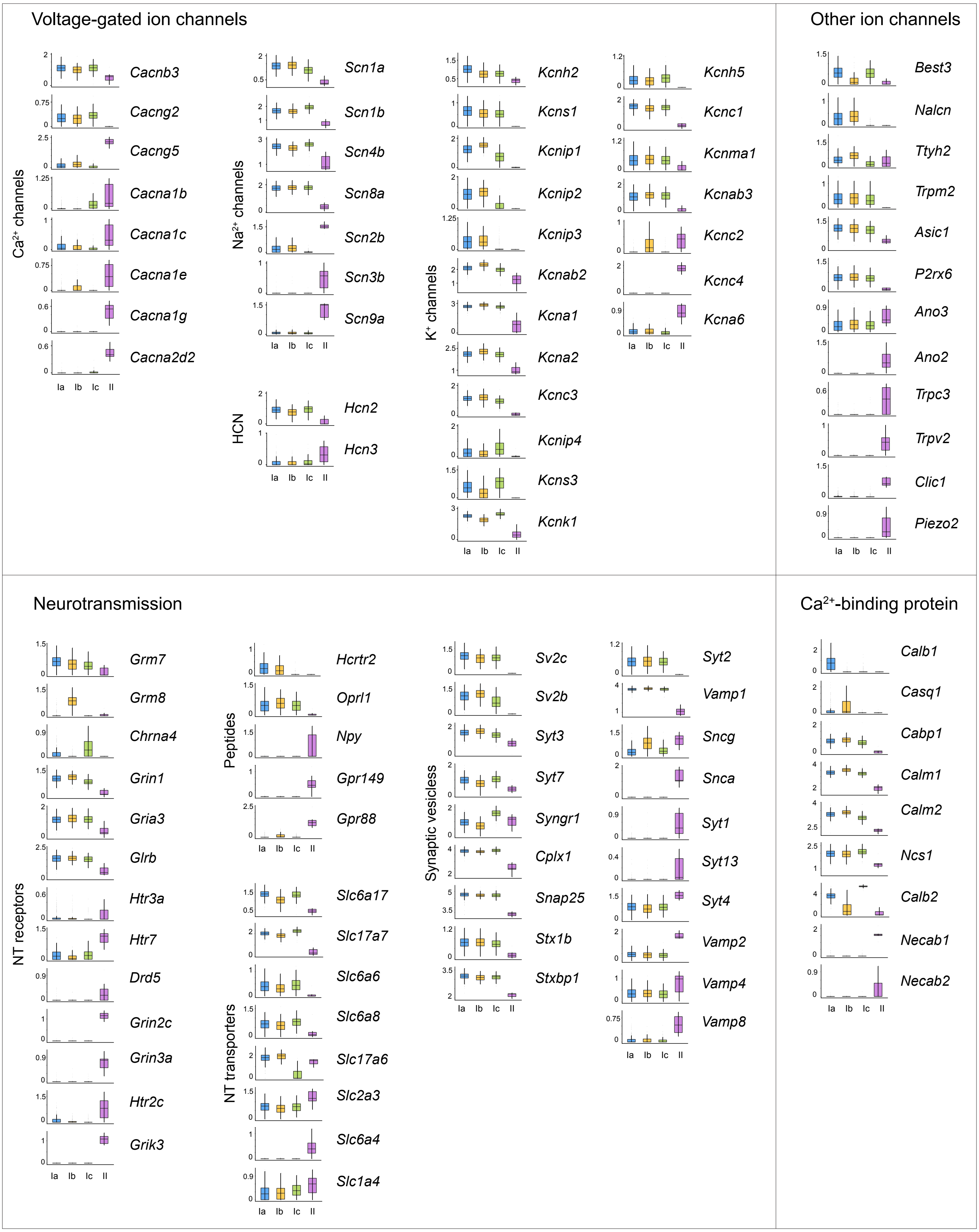

Supplementary Figure 3. Differential expression of genes related to synaptic transmission in adult SG neurons. Boxplots representing gene expression of genes related to Voltage-gated ion channels and to neurotransmission (Neurotransmitter receptors, peptides, neurotransmitter transporters, synaptic vesicle-related and calcium-binding proteins). The upper whiskers extend to the largest value no further than 1.5*IQR from the upper hinge, while the lower whiskers extend to the smallest value at most 1.5*IQR from the lower hinge. Dots represent the “outliers” beyond the range of whiskers.

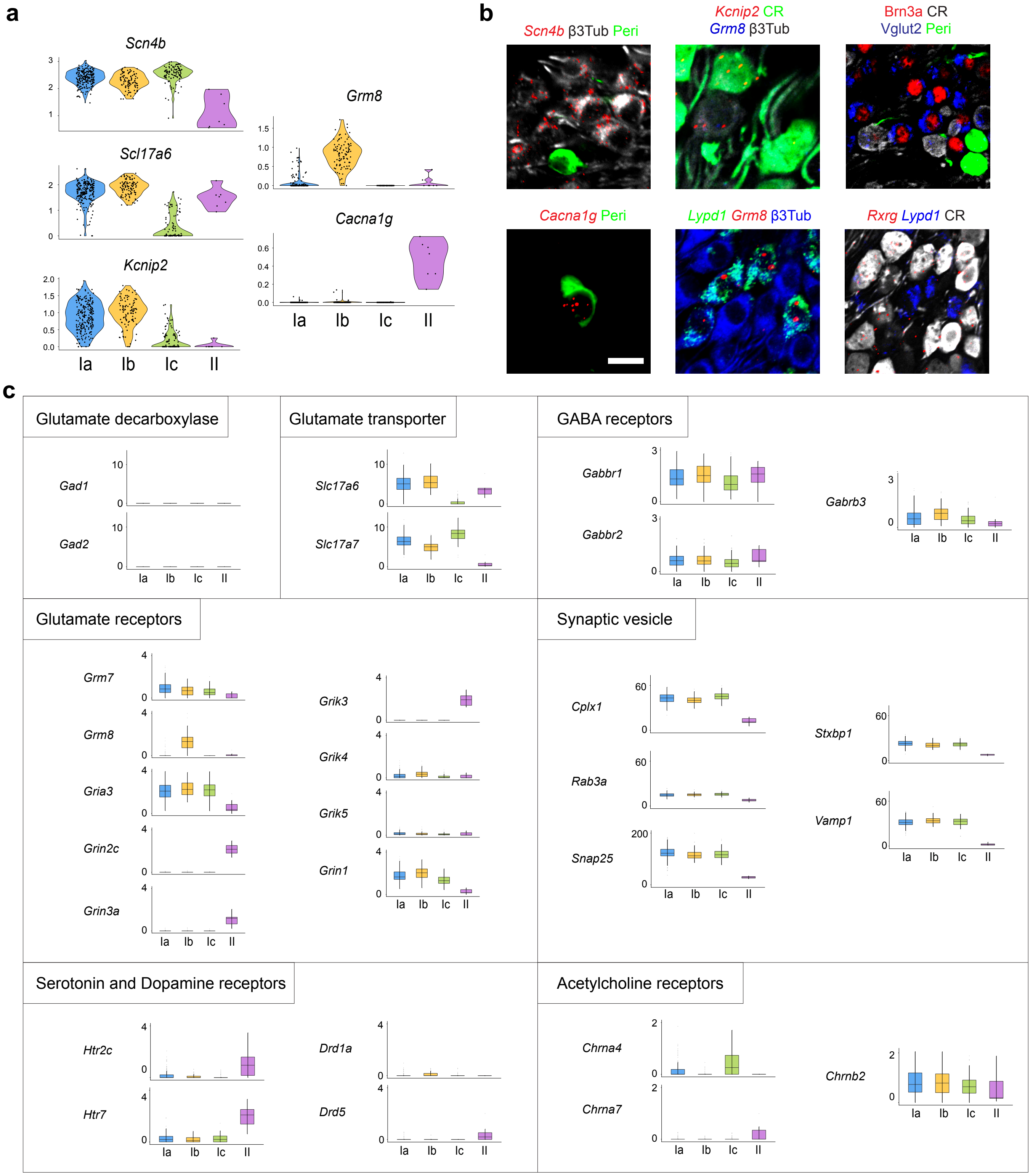

Supplementary Figure 4. Differential expression of key genes related to synaptic transmission in adult SG neurons. (**a**) Violin plots depicting expression of specific genes related to neurotransmission in our 4 populations of SG neurons in log-transformed scale. (**b**) *In vivo* validation of the expression and specificity of neurotransmitter related genes in (**a**) by immunohistochemical and fluorescent *in situ* hybridization on P21 SG sections. *Scn4b* is specific to all type I SG neurons, *Cacna1g* to type II neurons, *Grm8* to Ib neurons and Vglut2 (*Scl17a6*) and *Kcnip2* to Ia and Ib neurons. Note that co-localization on sections could never be observed for markers expressed in different populations of neurons in the scNRAseq data. (**c**) Boxplots representing gene expression of key genes related to glutamate transport, GABA, glutamate receptor, serotonin, acetylcholine receptor and synaptic vesicle. The upper whiskers extend to the largest value no further than 1.5*IQR from the upper hinge, while the lower whiskers extend to the smallest value at most 1.5*IQR from the lower hinge. Dots represent the “outliers” beyond the range of whiskers. Scale bar: 20 µm (**b**).

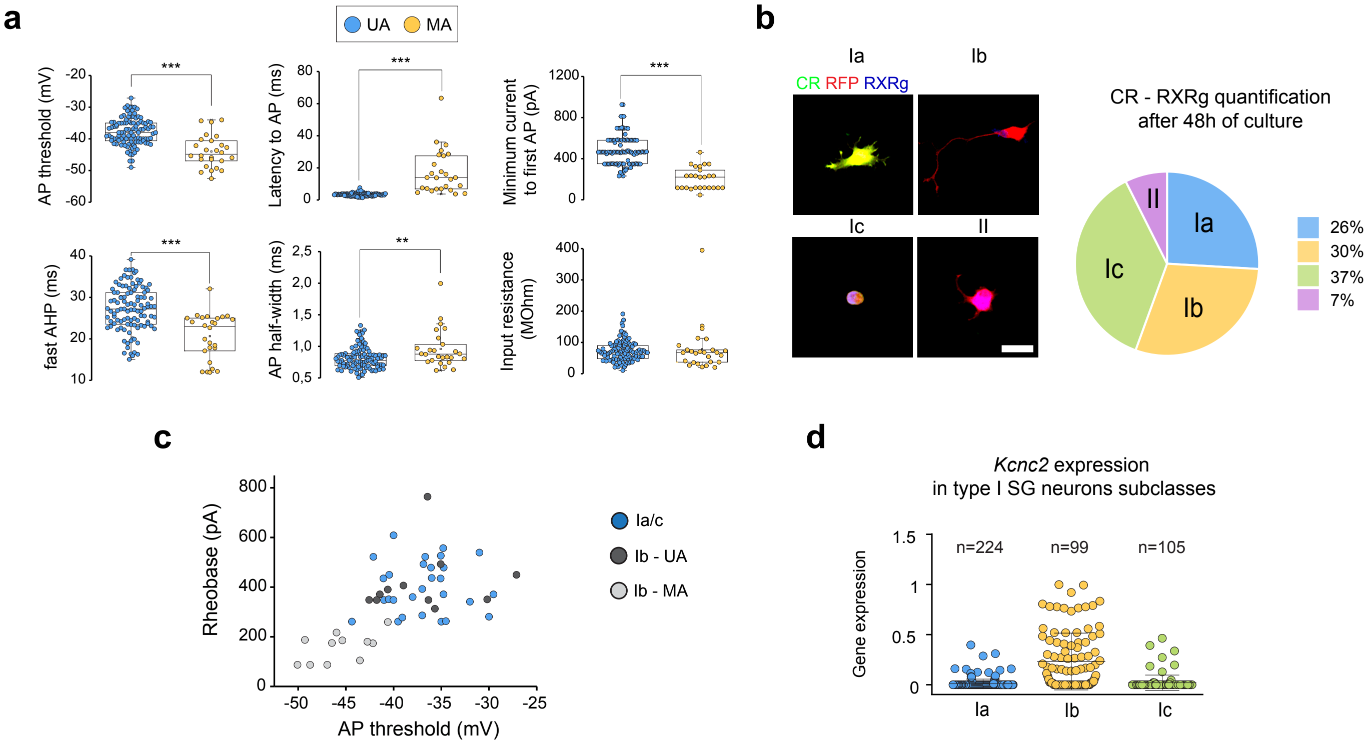

Supplementary Figure 5. Electrophysiological parameters of type I SG neurons. (**a**) Comparison of basic electrophysiological parameters using all recorded neurons (*n*=133) between UA and MA neurons. Data are presented as mean ± SEM (** $P \leq 0.01$, *** $P \leq 0.001$, *t*-test between UA and MA). (**b**) Post-hoc immunocytochemistry of SG neurons (from PV^{Cre};$R26^{TOM}$ mice) after electrophysiological recording, labeled by CR and RXRg followed by their quantification (*n*=150). Results illustrate that the proportion of neurons is maintained after 48h of culture. (**c**) Scatter plot of AP threshold versus Rheobase, with the labelling of Ia/c, Ib UA and Ib MA SG neurons. (**d**) *Kcnc2* expression among type I SG neurons (data from single-cell RNAseq), illustrating the sparse distribution among 50% of Ib neurons, and its relative absence in Ia and Ic neurons. Scale bar: 20 µm (**b**).

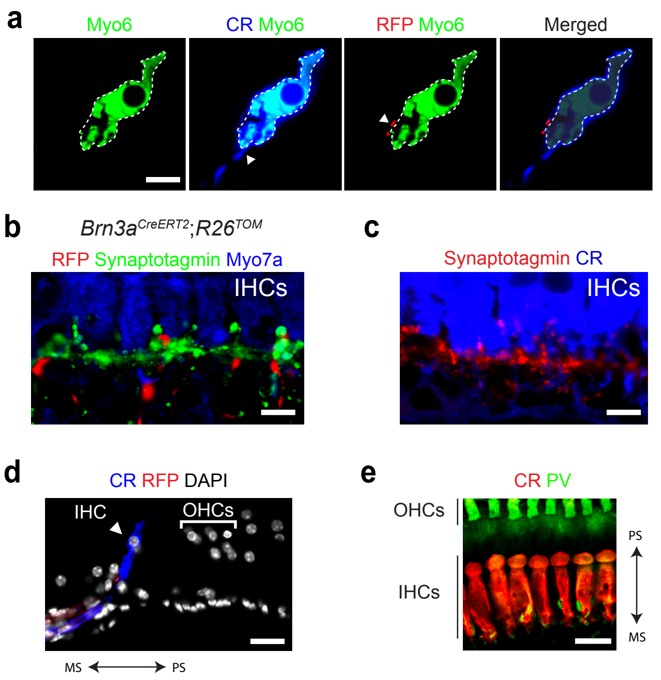

Supplementary Figure 6. Specific innervation pattern of type I neurons with IHC. (**a**) Analysis of the IHC innervation on cross section of P30 *Brn3a^{CreERT2};R26^{TOM}* mice (injected once with tamoxifen at P21) showing specific innervation of the modiolar side by RFP^{+} Ib fibers, while CR^{+} Ia/Ic fibers contact the pillar side of IHC. (**b**,**c**) Whole-mount immunostaining of cochlea from P30 *Brn3a^{CreERT2};R26^{TOM}* mice (**b**) or wild-type mice (**c**), illustrating the absence of co-localization between synaptotagmin positive efferents and RFP positive (Ib) or CR positive (Ia/Ic) afferents underneath the IHCs (labelled either by Myo7a or CR immunostaining). (**d**) Cross sections of an organ of Corti of a P30 *Brn3a^{CreERT2};R26^{TOM}* mice injected at P21 with tamoxifen, stained with CR, RFP and DAPI, and illustrating the modiolar side (MS, towards the spiral ganglion) and the pillar side (PS, towards the OHCs). (**e**) Whole-mount immunostaining of a wild-type P21 mouse cochlea, stained with CR and PV, and illustrating the modiolar (toward the spiral ganglion) and the pillar (towards the OHCs) sides. Note that in whole-mount, the view is form above the organ of Corti, with the organ compressed between the slide and a coverslip. In the merged panel, the IHC is shadowed to better visualize the innervation. Scale bars: 20 µm (**a**,**b**,**c**); 10 µm (**d**,**e**).

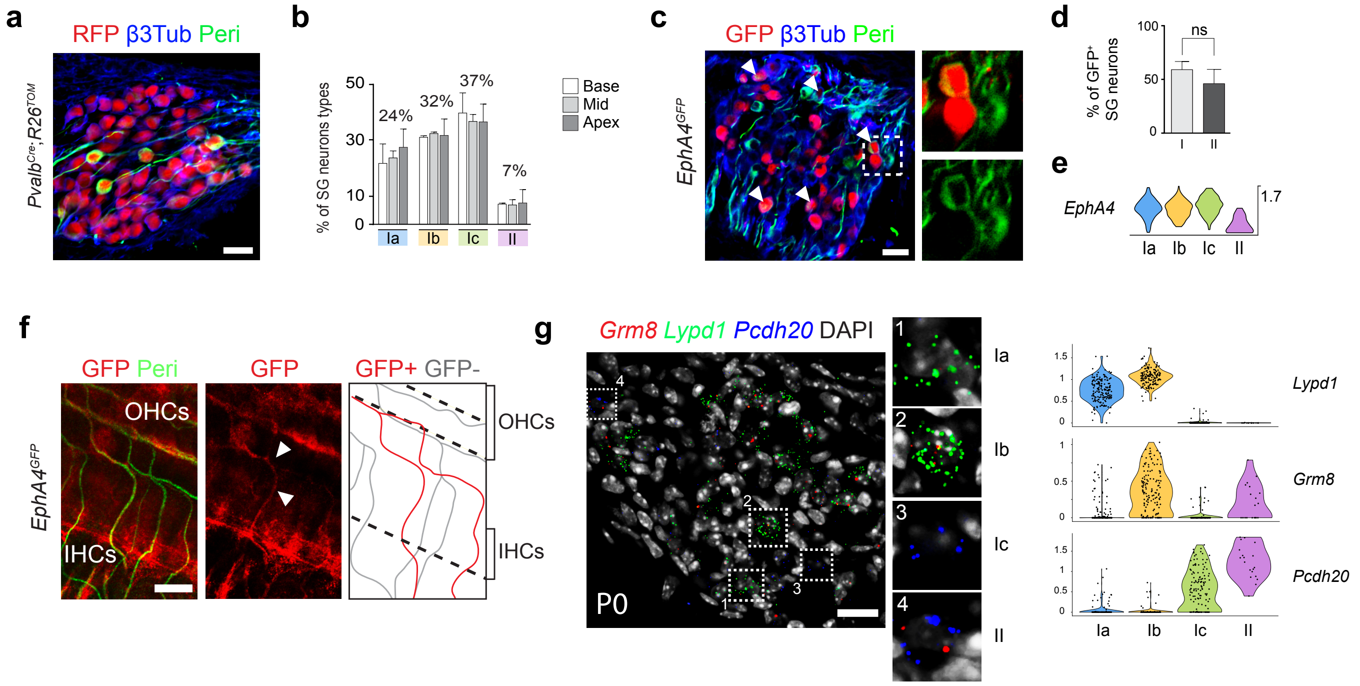

Supplementary Figure 7. Characterization of the expression profile of SG neurons at P3 and P0. (**a**) Immunostaining for RFP of SG section from *PV^{Cre};$R26^{TOM}$* mice, illustrating reporter expression in all neurons at P3. (**b**) Proportion of SG neuron subclasses along the tonotopic gradient (from base to apex) quantified by Runx1, Peri and CR expression at P3 (n = 3, data are represented as mean ± SEM). (**c**) Immunohistochemistry of SG section from *$EphA4^{GFP}$* mice at P3, showing GFP positivity in both type I (β3Tub$^+$/Peri$^-$) and II neurons (β3Tub$^+$/Peri$^+$). (**d**) Quantification of (**c**), showing that 57% of type I and 42% of type II SG neurons express GFP at P3. (n=3, P = 0.2198, unpaired t test, data are presented as mean ± SEM). (**e**) Violin plot showing *EphA4* expression in SG neurons (data from single-cell RNASeq), showing that type II neurons also express *EphA4*, albeit at lower level. (**f**) Immunostaining of flat-mounted SG from P3 *$EphA4^{GFP}$* mice, showing some GFP$^+$ type II fibers (Peri$^+$) crossing the tunnel of Corti to contact the outer hair cells (OHCs). (**g**) *In vivo* validation of the identified SG neuron subclasses at P0 by *in situ* hybridization using identified marker genes: *Lypd1* for Ia, *Grm8*/*Lypd1* for Ib, *Pcdh20* for Ic and *Grm8*/*Pcdh20* for type II neurons. Their expression in our scRNAseq data is represented by violin plots in log-transformed scale. Scale bars: 20 µm (**a**,**c**,**g**); 10 µm (**f**).

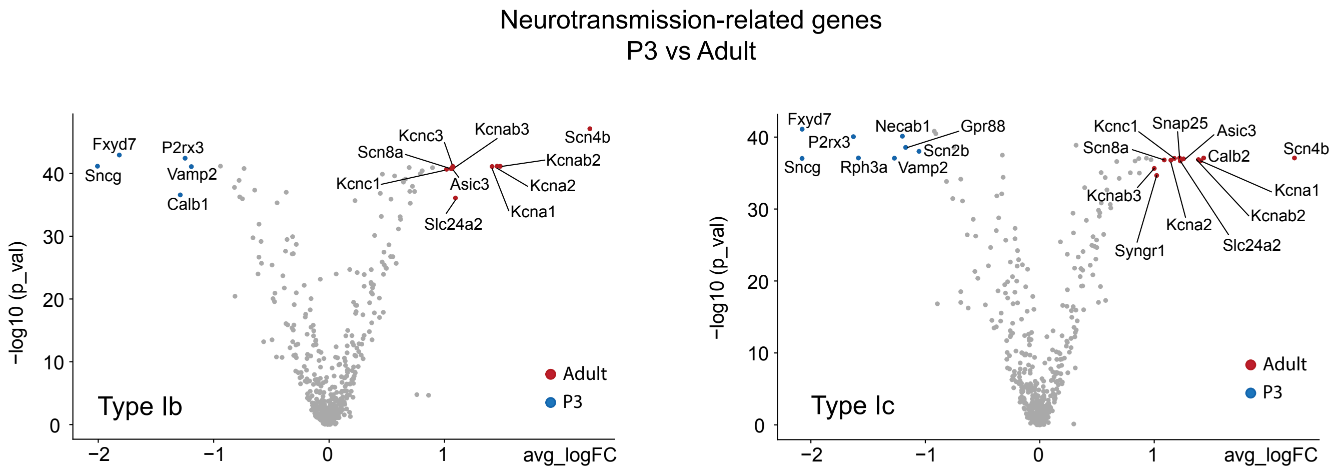

Supplementary Figure 8. Differential expression of neurotransmission related genes in P3 and adult SG neurons. Volcano plots of gene expression differences between adult and P3 SG neuron subclasses for Ib (left) and Ic (right) neurons. Genes differentially expressed in adult or P3 are marked by red or blue dots, respectively.

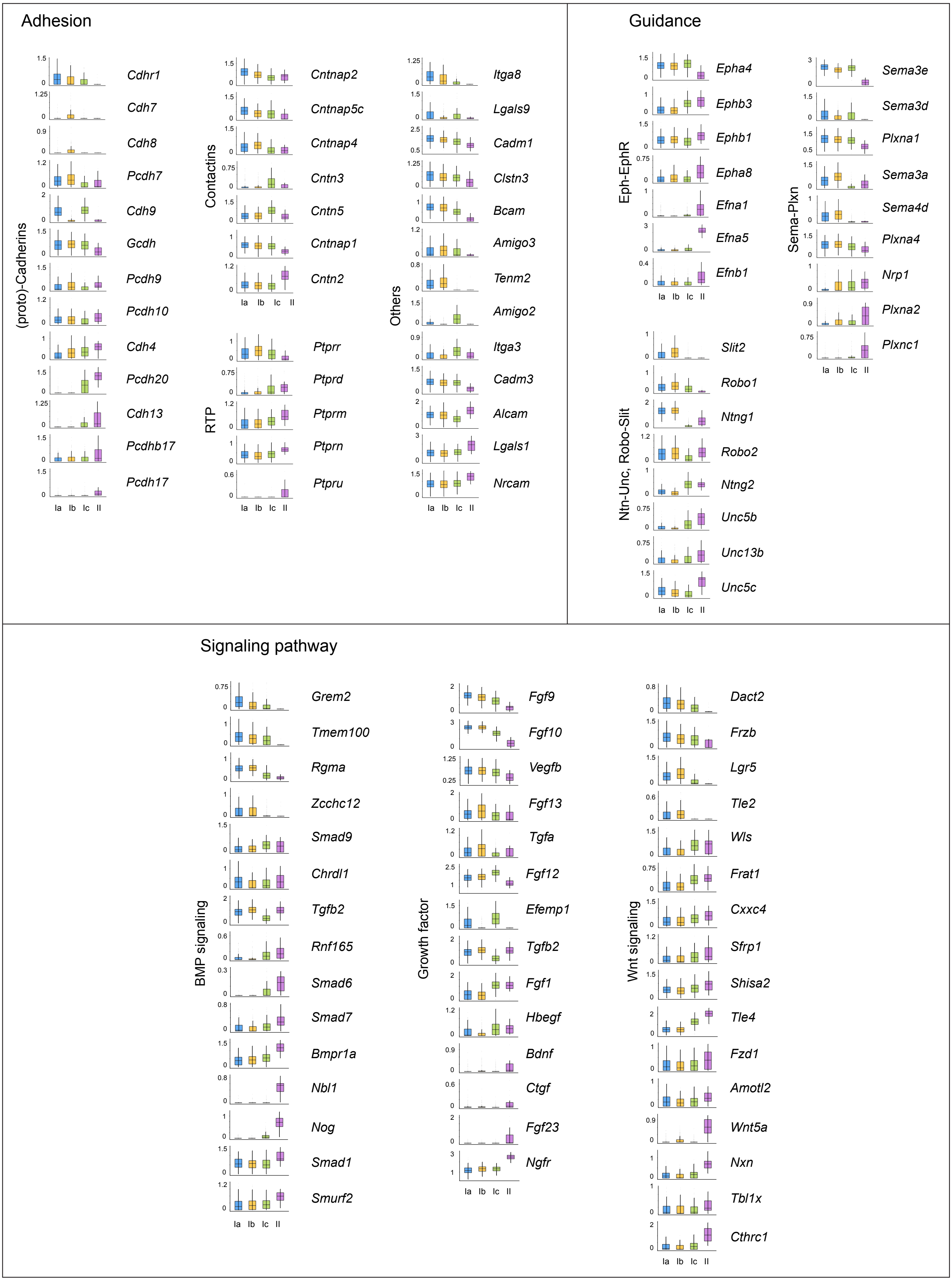

Supplementary Figure 9. Differential expression of genes related to adhesion, guidance and signaling pathway in P3 SG neurons. Boxplots representing gene expression of genes related to adhesion (Cadherin, Contactin, Protein tyrosine phosphatase (PTP) and other), to guidance (Ephrin, and Semaphorin) and to signaling pathways such as BMP signaling, growth factor and Wnt signaling. The upper whiskers extend to the largest value no further than 1.5*IQR from the upper hinge, while the lower whiskers extend to the smallest value at most 1.5*IQR from the lower hinge. Dots represent the “outliers” beyond the range of whiskers.

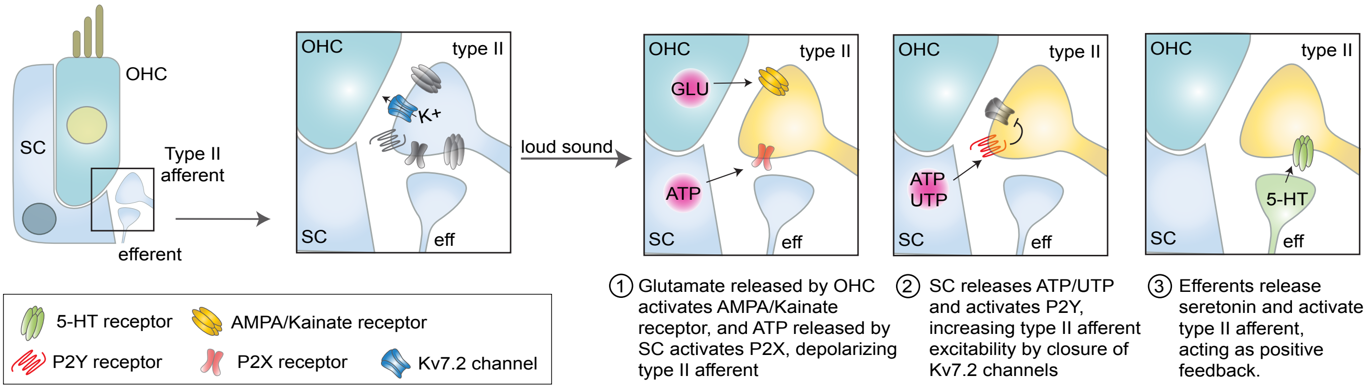

Supplementary Figure 10. Schematic of the hypothetical activation mechanisms of the type II SG neurons.

SC: supporting cell, CNS: central nervous system. Under control condition, M-current, mediated by the opening of K_v7.2 channel, is active and raises the threshold of firing, and the probability of glutamate release by OHCs is low: type II neurons are silent. Following exposure to loud sound, the number of activated OHCs increases, together with the probability of glutamate release and the synaptic inputs in type II neurons through AMPA/kainate receptors activation. In parallel, ATP released by damaged SCs activates P2X receptors in type II neurons. Both the activation of AMPA/kainate and of P2X receptors lead to spike generation in type II neurons and signal transmission (**1**). In parallel, ATP and UTP released by SCs can also activate P2Y receptors in type II neurons, which, via IP3-Ca^{2+} signal, inhibit M-current. This results in a decrease of the threshold current required to fire an action potential and sensitizes further the type II neurons to synaptic inputs generated by activation of AMP/kainate and P2X receptors (**2**). A positive central feedback loop through the release of serotonin (5-HT) by medial olivocochlear efferents and the activation of 5-HT receptors in type II neurons could also contribute to type II neurons activation or sensitization (**3**).

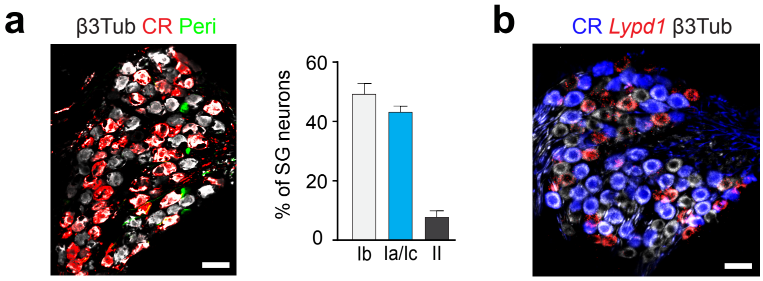

Supplementary Figure 11. Quantification of SG neurons subclasses in adult rat. (**a**) Immunohistochemistry and quantification of SG neurons subclasses using CR, peripherin and β3-tubulin antibodies (*n*=3; data are presented as mean ± SEM). (**b**) ISH (RNAscope) for *Lypd1* and immunostaining for CR and β3-tubulin confirm mutually exclusive expression of CR (Ia/Ic neurons) and *Lypd1* (Ib neurons) in adult rat SG section. Scale bar: 20 µm (**a**,**b**).

Supplementary Table 5. Antibodies used in the study

Antibodies	Source	Concentration	Identifier (Cat#)
IF: chicken anti-RFP	Rockland	1:250	600-401-379
IF: goat anti-Prph	Everest Biotech	1:10 000	EB12405
IF: mouse anti-β3tub	Promega	1:500	G712A
IF: rabbit anti-Th	Pel Freez	1:1000	P40101-150
IF: rabbit anti-Brn3a	Eric Turner lab	1:500	Quina et al., 2012[4]
IF: rabbit anti-Runx1	Thomas Jessell lab	1:500	Chen et al., 2006[5]
IF: mouse anti-Calb2	Swant	1:500	CB-300
IF: rabbit anti-Calb2	Swant	1:500	6B3
IF: rabbit anti-Myo6	Proteus Bio	1:500	25-6791
IF: rabbit anti-Myo7a	Proteus Bio	1:500	25-6790
IF: rabbit anti-Rxrg	Assay Biotechnology	1:500	C0316
IF:mouse anti-synaptophysin	Millipore	1:1000	MAB329
IF: mouse anti-LacZ	Promega	1:500	Z378B

Supplementary Table 6. RNAscope probes used in the study

RNAscope probes	Source
Mm - Plk5	ACDBio
Mm - Cacna1g	ACDBio
Mm - Rxrg	ACDBio
Mm - Calb1	ACDBio
Mm - Lypd1	ACDBio
Mm - Pcdh20	ACDBio
Mm - Gabrg2	ACDBio
Mm – Scn4b	ACDBio
Mm – Kcnip2	ACDBio
Mm – Grm8	ACDBio

Supplementary Table 7. GO terms used to retrieve genes related to certain functions

GO id	Term
0003700	DNA binding transcription factor activity
0005216	Ion channel activity
0005509	Calcium ion binding
0005746	Mitochondrial respiratory chain
0005856	Cytoskeleton
0006754	ATP biosynthetic process
0006811	Ion transport
0006836	Neurotransmitter transport
0007155	Cell adhesion
0007156	Homophilic cell adhesion via plasma membrane adhesion molecules
0007157	Heterophilic cell adhesion via plasma membrane adhesion molecules
0007218	Neuropeptide signalling pathway
0007269	Neurotransmitter secretion
0008021	Synaptic vesicle
0008076	Voltage-gated potassium channel complex
0030594	Neurotransmitter receptor activity
0007411	Axon guidance
0008083	Growth factor activity
0016055	Wnt signalling pathway
0030509	BMP signalling pathway
0060070	Canonical Wnt signalling pathway

Supplementary References

1 Tong, L. *et al.* Selective deletion of cochlear hair cells causes rapid age-dependent changes in spiral ganglion and cochlear nucleus neurons. *J Neurosci* **35**, 7878-7891 (2015).

2 Kirjavainen, A. *et al.* Prox1 interacts with Atoh1 and Gfi1, and regulates cellular differentiation in the inner ear sensory epithelia. *Dev Biol* **322**, 33-45 (2008).

3 Chonko, K.T. *et al.* Atoh1 directs hair cell differentiation and survival in the late embryonic mouse inner ear. *Dev Biol* **381**, 401-410 (2013).

4 Quina, L.A., Tempest, L., Hsu, Y.W., Cox, T.C. & Turner, E.E. Hmx1 is required for the normal development of somatosensory neurons in the geniculate ganglion. *Dev Biol* **365**, 152-163 (2012).

5 Chen, C.L. *et al.* Runx1 determines nociceptive sensory neuron phenotype and is required for thermal and neuropathic pain. *Neuron* **49**, 365-377 (2006).
