# Supplementary material for: Neuronal heterogeneity and stereotyped connectivity in the auditory afferent system
Source: Nat Commun. 2018 Sep 12;9:3691. doi: 10.1038/s41467-018-06033-3 (PMC6135759; doi:10.1038/s41467-018-06033-3)
Supplement: Supplementary file 2 — Peer Review File [file 41467_2018_6033_MOESM2_ESM.pdf]

Reviewers' comments:

Reviewer #1 (Remarks to the Author):

The study by Charles Petitpré and colleagues identifies three novel classes of auditory afferent neurons with unique molecular and functional characteristic and connectivity patterns. The question of whether distinct neuronal subtypes convey the brains auditory inputs is an extremely important one. As stated by the authors "spiral ganglion (SG) neurons of the inner ear cochlea convey all brain's auditory inputs, yet the cellular and molecular complexity necessary to decode the various acoustic features in the SG has remained unresolved". Currently, SG neurons (SGN) are classified into type I and type II SGNs. However, electrophysiological data indicates that type I SGNs consist of at least two different populations of neurons, which based on their threshold to acoustic stimulation have been classified as high threshold (HT) and low threshold fibers (LT).

Here, using unbiased single cell RNA sequencing of functional mature SGN, the authors demonstrate the existence of four distinct SGN sub- types. The authors find that ~7% of SGNs are type II SGNs, and that the remaining ~93% of type I SGNs represent three distinct populations, with 26% type Ia, 24% type Ib and 43% type Ic SGNs. The authors catalog unique SGN subtype-specific molecular signatures providing novel insights into their function, synaptic partners and metabolic demands. Going a step further, performing whole cell recording on dissociated type I SGNs and peripheral projection tracing experiments, the authors demonstrate that previously described HT and LT SGNs represent distinct sub-types, with HT neurons consisting mainly of type Ib SGNs and LT consisting of type Ia and type Ic SGNs. Finally the authors provide evidence that the specification into the four SGN sub-classes may occur independently of neuronal activity.

These findings are highly significant. The identification and characterization of three novel subtypes of SGNs will have a lasting impact on the auditory field and the broader field of sensory biology and neuroscience. Overall the major claims of the study are well supported by the provided data, the authors use state of the art methodology and experiments and outcomes are well described and discussed. I have only few suggestions for improvement.

Minor:
1) The authors provide evidence that four distinct SGN subtypes already exist at stage P3. Interestingly, the relative proportions of type Ib and type1c SGNs are different in P3 vs adult animals (P3 24% type Ia, 32% type Ib, 37% type Ic; adult: 26% type Ia, 24% type Ib and 43% type Ic SGN. Are P3 type- Ib SGNs converting into type Ic SGNs at later stages?
2) The authors state that ".. the four neuron types could be already identified at P0 (data no shown)". Data not shown is not acceptable -authors need to provide the data.
3) The authors state that the proportion of cell types is constant along the tonotopic axis. However based on information provided in the figure legend "proportion of SG neurons types along the tonotopic gradient (from base to apex) quantified by Runx1 and CR expression.." only two markers Runx1 and CR were used –these markers mark differentially SGN type-type I neurons. How were SGN type II neurons quantified?
4) The authors conduct electrophysiological recordings that reveal that 20% of all type I

SGNs are multiple spikes accommodating (MA) cells and that 80% are unitary spike accommodating (UA) cells. Based on post-hoc staining the authors then conclude that all type1a, type1c neurons are UA neurons, whereas 50% of type 1b SGNs are MA cells (which by my estimation corresponds to 12.9% of all type I SGNs). What happened to the remaining 7.1% of MA cells? What is their identity? Clarification is needed.
5) The electrophysiological recordings also suggest that type 1b SGNs may consist of 2 subtypes of neurons (UA and MA); the authors re-analyze single cell expression data and present Kcnc2 (Kv3.2) as potential candidate gene that may drive the distinct properties of MA cells –Is Kcnc2 the only gene with " contrasted expression"? And if so could driving Kcnc2 expression in dissociated SGNs convert a UA into a MA cell? These open questions should be addressed.
6) The electrophysiological characterization of dissociated type 1 SGNs would be strengthen by performing it on genetic labeled SGN cells (e.g. Brn3a-CreERT2;R26-TOM for type1b SGNs).
7) Minor editing issues:
Some abbreviations are not defined- e.g. AP, IHC, ISI
tSNE plot misses axis label

Reviewer #2 (Remarks to the Author):

Neurons in the spiral ganglion (SG) in the cochlea relay auditory information from the hair cells (HCs) to the brain. Two types of SG neurons, type I and type II, have previously been identified, which connect to the inner hair cells (IHCs) and outer hair cells (OHCs), respectively. The diversity in the biophysical properties of type I SG neurons has been hypothesized to underlie the coding of diverse aspects of auditory information, but the molecular and cellular basis of this diversity is not fully understood. In this manuscript, the authors performed single-cell RNA sequencing using the Smart-Seq2 platform to profile ~500 neurons from the adult and P3 SG, respectively. They classified the neurons into four molecularly separable clusters that represented the type II neurons and three novel subtypes of type I neurons (Ia, Ib, and Ic), identified rich molecular markers for each neuronal type, and showed that these molecular distinctions were established before the onset of hearing independent of sound stimulation. They performed electrophysiological characterizations on cultured SG neurons and found that the three subtypes of type I neurons may have different properties; Ia and Ic neurons were unitary spike accommodating neurons and Ib neurons were comprised of both unitary and multiple spike accommodating neurons. They further showed that the afferent terminals of the three type I subclasses were spatially separated; Ia and Ic neurons innervate the pillar side whereas Ib neurons innervate the modiolar side of the IHCs. These findings hold the potential of advancing our understanding of the molecular and cellular basis for the functional diversity of SG neurons and could be of general interest to the field. However, a number of points need to be addressed.

Major points:
1. One main concern is the markers used for labeling different subclasses of type I neurons

in a number of experimental characterizations. In Fig. 1 and Fig. S1 (quantification of cell number), Fig. 4 (electrophysiological recording), and Fig. 5 (afferent labeling), the authors used CR (CALB2) to label Ia and Ic neurons. However, according to the results shown in Fig. 1f, Calb2 was not only expressed in Ia and Ic neurons but also showed considerable expression in Ib neurons. How could the authors be sure that the CR+ cells only contained Ia and Ic neurons but not Ib neurons? Also, in Fig. 5, the authors used Pou4f1 as a marker for Ib neurons. Based on Fig. 1f, this gene was also expressed at a considerable level in Ia neurons. Again, how did the authors ensure labeling specificity? Fig. 5b showed that 95% of Pou4f1+ neurons were Lypd1+ (an Ib neuron-specific marker). However, based on the image shown, less than 50% of Lypd+ neurons were Pou4f1+. This suggested that a subpopulation of Ib neurons were not labeled by Pou4f1. Have the authors examined the innervation pattern of this subpopulation? In general, why not use markers that were restricted to a single subtype, e.g. Calb1 for Ia, Lypd1 for Ib, and Trim54 for Ic?

2. For the validation of subtype markers presented in Fig. 1g and Fig. 6c, no quantification was provided. The authors should quantify the overlap between markers that were predicted to label the same or different subtypes.

3. The authors proposed that the different expression levels of the K+ channel Kv3.2 in different Ib neuron subpopulations may contribute to their different electrophysiological properties (UA vs. MA type). This is an interesting hypothesis and should be directly tested by post-hoc immunostaining of Kv3.2 (as the authors have done for CR) to examine whether there is indeed a correlation between Kv3.2 expression level and whether an Ib neuron is UA or MA type.

Minor points:
1. For the gene set enrichment analysis, It should be indicated what genes were used as background for the analysis; only the expressed genes should be used. Also, it should be indicated whether the p values have been corrected for multiple testing.

2. Fig. S3b and S3c were mislabeled.

3. In Fig. 6i and Fig. S5f, do the plots show all genes or only gene families related to neurotransmission? Were the p values corrected for multiple testing? These were not clear from the text or figure legends.

4. In the Abstract, "exhaustive transcriptional catalog" seems an overstatement, given that only hundreds of cells have been profiled. Additional molecular subtypes could be identified when more cells are sampled. Indeed, the electrophysiological experiments in this study suggested that the Ib neurons may be further comprised of subpopulations of different electrophysiological properties.

Reviewer #3 (Remarks to the Author):

Review of Petipre and Wu et al

The manuscript by Petipre, Wu and colleagues was a pleasure to read and will provide an excellent resource to investigators in the auditory field. Their study takes advantage of relatively new single cell capture technology and RNA sequencing and delineates, at the molecular level, different classes of SGNs. This study is comprehensive and includes cell sorting and sequencing, histological and morphological analyses and electrophysiology. In general, the manuscript is of high quality, but I have several suggestions to help strengthen the manuscript. Most of my concerns are related to important controls and quantification and should be relatively easy to address.

Major concerns:

1. Some of the most important data points can be found in the violin plots. In the figures shown, the violin plots are far too small. The y axes are compressed, and they are difficult to interpret in a quantitative sense. For all of the violin plots shown, the dots that indicate the individual cells that contributed to the width of the violin are needed. The dots help show the raw data and allow the reader to compare between groups.

2. Most of the histology is limited to the core set of factors that were discovered that define each group. Why not add some in situ hybridization experiments for select factors within those groups? As it stands, Figures 2, 3, and 7 is all data analysis. For example, the authors should show in situ hybridization data for one or two of their "functional signatures" genes as proof of concept and show that the cells with high levels of those genes appear at the expected frequency.

3. The authors should show some proof that there is no contamination by hair cells into the SGN sorts. The use of PV^Cre brings this possibility. I became concerned about this seeing that VGlut1 and Piezo2 were noted as SGN markers, and these factors are known to be expressed by hair cells. Apparently, the sensory domain is mechanically separated from the SGNs for their sorts, but some evidence showing that the tdTomato population that was used for the sort did not contain hair cells would be beneficial. The presence of Prox1, for example, engenders confidence that the cells are mostly SGNs, but Prox1 is known to be expressed transiently in hair cells (albeit at much earlier stages).

4. For all of these new markers shown by either immunofluorescence or RNAscope, there is no discussion or demonstration of staining specificity. Indeed, the lack of staining in neighboring cells acts as an "internal control," but some information on the epitopes for these factors and the ways that their specificity was ascertained would be helpful, especially for anyone wanting to replicate their findings.

5. All of the significant p-values can be found in the supplementary Excel spreadsheet. This is very unsatisfying. In addition, the colorful squares are very nice and beautifully arranged in the figures, but the reader is left with no sense of the raw data or significance values. I would recommend that the authors devise a way, for at least some of the important genes, to display statistical significance.

6. For figure 5 on the spatial segregation of the type I SGNs, the authors need to quantify

the differences they are describing. This analysis needs to be far more rigorous. As it stands the reader has no reason to be confident in the illustration in 5g. What criteria did the authors use to define "pillar" vs "modiolar?" What neighboring cells were used as landmarks?

Minor points:
1. For the micrographs shown, the ages of the mice should be indicated.
2. The first sentence of the Abstract needs to be revised. The possessive "brain's" doesn't really fit.

Response to reviewers' comments:

Reviewer #1

We are very glad that the reviewer finds our study of high significance and that (s)he finds that our results support the conclusions, with only minor issues to be addressed. Please find below a response to the criticism and an outline on the modifications introduced into the manuscript to fully address the raised issues.

1) *The authors provide evidence that four distinct SGN subtypes already exist at stage P3. Interestingly, the relative proportions of type Ib and type1c SGNs are different in P3 vs adult animals (P3 24% type Ia, 32% type Ib, 37% type Ic; adult: 26% type Ia, 24% type Ib and 43% type Ic SGN. Are P3 type- Ib SGNs converting into type Ic SGNs at later stages?*

We thank the reviewer to raise this point, which need clarification in the main text. We have analyzed in details our data and have not found any statistical difference in the proportion of each subclass of neurons between P3 and adult stages, from base to apex ($P > 0.05$, comparing Ia P3 with Ia adult, and so on for all subclasses, see Table below). We performed new immunostaining experiments using an antibody against Brn3a (*Pou4f1*), combined to CR and peripherin staining to confirm the proportion of each subclass of neurons (Ia, Brn3a$^+$/CR$^+$; Ib, Brn3a$^+$; Ic, CR$^+$; II, Peri$^+$). Results show 29% of Ia, 28% of Ib, 38% of Ic and 5% of II (see the new Supplementary Fig. 1f), which also does not show any statistical difference with the proportion of each subclass found at P3 ($P > 0.05$). This is now clarified in the main text, page 14, 1st paragraph of “Neuronal diversity in the cochlea is already established at birth”.

Comparison of the proportion of each SG neuron subclass between P3 and adult:

	P3	mean	Adult	mean	significance
Base	Ia	21,79887	Ia	21,84915	ns
	Ib	31,14358	Ib	26,11127	ns
	Ic	39,81392	Ic	43,75844	ns
Middle	Ia	23,7631	Ia	22,44386	ns
	Ib	32,48138	Ib	27,99083	ns
	Ic	36,80854	Ic	42,20126	ns
Apex	Ia	27,55331	Ia	27,12934	ns
	Ib	31,79579	Ib	24,95384	ns
	Ic	36,64367	Ic	42,0321	ns

2) *The authors state that “.. the four neuron types could be already identified at P0 (data no shown)”. Data not shown is not acceptable -authors need to provide the data.*

We apologize for only citing this observation in our original manuscript. Our data on the identification of the 4 neuron types at birth has been now added in Supplementary Figure 7g. The figure shows the expression of *Grm8* (Ib and II, data from scRNAseq at P3 Fig. 6b, Suppl. Fig. 7g),

Lypd1 (Ia and Ib specific at early postnatal stages, see Fig. 6b and c, Suppl. Fig. 7g, data from scRNAseq and RNAscope at P3) and *Pcdh20* (Ic and II specific, data from scRNAseq and RNAscope at P3, see Fig. 6b and c, Suppl. Fig. 7g) on cochlea sections from P0 animals (WT). Thus, similarly to the P3 data, Ia are *Lypd1*$^{+}$, Ib are *Lypd1*$^{+}$/*Grm8*$^{+}$, Ic are *Pcdh20*$^{+}$ and II are Grm8^{+}/Pcdh20^{+} at P0. The figure legends of the Supplementary Fig. 7 and the main text have been changed accordingly.

3) *The authors state that the proportion of cell types is constant along the tonotopic axis. However based on information provided in the figure legend "proportion of SG neurons types along the tonotopic gradient (from base to apex) quantified by Runx1 and CR expression.." only two markers Runx1 and CR were used –these markers mark differentially SGN type-type I neurons. How were SGN type II neurons quantified?*

We thank the reviewer to notify us the absence of information on the quantification of the type II neuron population. For this, we used anti-peripherin staining to specifically label type II neurons. Note that Runx1 and calretinin positive cells never co-localized with peripherin, confirming the scRNA sequencing data and the specificity of peripherin expression in type II neurons from P1 onward (Hafidi, Brain Res., 1998; Lallemend *et al*., Neurosci., 2007; Huang *et al*., Development, 2007). This information on peripherin staining has been now added in the legend of the Figures 1, 6 and of the Supplementary Figure 1.

4) *The authors conduct electrophysiological recordings that reveal that 20% of all type I SGNs are multiple spikes accommodating (MA) cells and that 80% are unitary spike accommodating (UA) cells. Based on post-hoc staining the authors then conclude that all type1a, type1c neurons are UA neurons, whereas 50% of type 1b SGNs are MA cells (which by my estimation corresponds to 12.9% of all type I SGNs). What happened to the remaining 7.1% of MA cells? What is their identity? Clarification is needed.*

We realize that the text might not have been very clear when comparing the data without and with post-hoc immunostaining. Here is a more detailed description of the analysis. The electrophysiological recordings were performed on 133 cells, out of which 107 cells (80%) were UA and 26 cells (20%) were MA. From these neurons, only 50 cells could accurately be analyzed by immunostaining. Of these 50 cells, 22 were identified as Ib, with 11 Ib being UA, the other Ib being MA. 11 Ib neurons that are MA represent approximately 20% of all analyzed 50 cells. And the remaining 80% are indeed UA cells.

We have now clarified it within the main text. "Post-hoc immunostaining on 50 cells revealed that all Ia and Ic neurons (28 TOM^{+}/CR^{+} neurons in a *PV*^{Cre};*R26*^{TOM} context) corresponded to UA type (Fig. 4a-b and e-g, Supplementary Fig. 3b), while the Ib population (22 TOM^{+}/CR^{-} neurons) was equally comprising either UA (11 cells) or MA type (11 cells)."

Please note that the proportion of each subpopulation of type I neurons between the *in situ* data (24% of Ib neurons) and the *in vitro* recorded neurons (~40% of Ib neurons) are difficult to be compared, because of the time in culture, the conditions used and the success or failure to patch single sensory neurons, which are all conditions or characteristics of the experiment that could bias the proportion of recorded neurons in each subclass.

5) *The electrophysiological recordings also suggest that type 1b SGNs may consist of 2 subtypes of neurons (UA and MA); the authors re-analyze single cell expression data and present Kcnc2 (Kv3.2) as potential candidate gene that may drive the distinct properties of MA cells –Is Kcnc2 the only gene with " contrasted expression"? And if so could driving Kcnc2 expression in dissociated SGNs convert a UA into a MA cell? These open questions should be addressed.*

In search for the genes which change together with *Kcnc2* expression, we have used an automated approach and performed Differential Expression Analysis (using Deseq2 R package) to identify genes differentially expressed in Kcnc2$^+$ or Kcnc2$^-$ populations (positive cells have *Kcnc2* expression level > 0.1 in our dataset, the negative cells have level = 0). No gene was found to follow *Kcnc2* expression.

In a different parallel approach to search for genes that could contribute to the MA and UA distinction of the Ib neurons independently of *Kcnc2* expression profile, we have plotted heatmaps showing expression level of genes associated with neurotransmission in all cells, looking for genes which are expressed in about half population of Ib neurons and not at all in Ia/Ic neurons (for genes specific to MA type Ib neurons), or for genes expressed in all Ia/Ic neurons and only in about 50% of Ib neurons (for genes specific to UA type Ia/Ic and UA type Ib neurons). Apart from *Kcnc2*, only two other genes showed a contrasted expression: *Kcns3* and *Best3* (see Figure below).

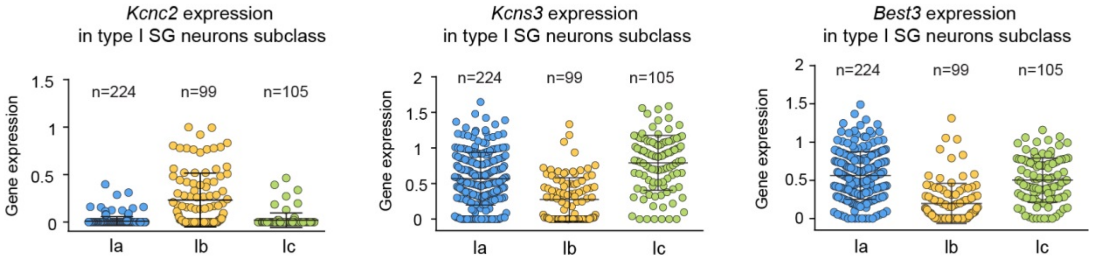

Figure. *Kcns3* and *Best3* expression compared to *Kcnc2* expression among type I SG neurons (data from single-cell RNAseq). Note the sparse distribution of *Kcnc2* among 50% of Ib neurons, and its relative absence in Ia and Ic neurons. At the opposite, *Kcns3* and *Best3* are expressed in most Ia and Ic neurons (only 10-20 cells do not express them in the Ia and Ib neurons, which represents about less than 10% of their population), while showing contrasted expression in Ib neurons, in which 50% of the population (50 cells our of 99 cells) do not express those genes.

Best3 is coding for the integral membrane protein bestrophin-3, which functions as an intracellular calcium-activated chloride channel (and permeable to glutamate and GABA) on the cellular membrane of astrocytes and of some neurons and that is not voltage-dependent. It most likely does not participate in the generation or shape of action potential. Instead, it would be involved in the formation of calcium stores within the cell and/or release of glutamate (Oh and Lee, Exp. Neurobiol., 2017). *Kcns3* is coding for $K_v9.3$, which are unable to form functional channels but heterotetramerize with $K_v\alpha 2$ family members (genes *Kcnb1* and *b2*) to form functional channels (Bocksteins E., JGP, 2016). *Kcnb1/2* are however not expressed in cochlear neurons in our data set. In conclusion, to the best of our analysis, only *Kcnc2* seems to be a good candidate to function in a subpopulation of Ib neurons. Further work will be needed both *in vitro* and *in vivo* to study the function of this gene in the physiology of type Ib neurons and in hearing. This has now been addressed more clearly in the main text (second last paragraph of the result section on electrophysiology): "To the best of our analysis, *Kcnc2* was the only candidate gene in our neurotransmission-related gene dataset to show this contrasted expression in type Ib neurons. In view of the importance of K_v3 channels in the

regulation of the firing properties of neurons (Kaczmarek and Zhang, 2017), it will be interesting to assess *in vitro* and *in vivo* the role of $K_v3.2$ in the physiology of type Ib neurons and in hearing."

6) *The electrophysiological characterization of dissociated type 1 SGNs would be strengthen by performing it on genetic labeled SGN cells (e.g. Brn3a-CreERT2;R26-TOM for type1b SGNs).*

This experiment was originally planned. However, the $Brn3a^{CreERT2};R26^{TOM}$ mouse line can only be used at low efficiency if one wishes to target only or preferentially the Ib population. With one injection of tamoxifen to activate recombination in target cells, mostly Ib neurons are labelled (94% of RFP^+ type I neurons are Ib neurons then), but with an efficiency of about 25% at best. In these conditions, since Ib neurons represent 25% of all cochlear neurons, TOM^+ Ib neurons represent only ~6% of all neurons. These neurons being of two types electrophysiologically (MA/UA), only 3% of neurons would be eventually TOM^+ MA type Ib neurons. However, this percentage is a large over-representation since recombination efficiency using so little tamoxifen can be much lower. This percentage rose to ~70% of labelled Ib neurons with 2 injections of tamoxifen with one day interval, however leading also in parallel to a much higher recombination within the Ia population (from 6% to 30%). So, our low recombination strategy was useful for sparse labelling of neuronal projections underneath the hair cells, but is inadequate for electrophysiological studies where a large number of labelled neurons of a single type would be required. The utility but also limitations of our sparse labelling strategy have now been added within the main text and Material and Methods section under the subtitle "Tamoxifen-induced sparse labelling".

7) *Minor editing issues:*
Some abbreviations are not defined- e.g. AP, IHC, ISI
tSNE plot misses axis label

This has been corrected in the text, and axis labels have been added to the tSNE plots.

Reviewer #2

We thank the reviewer for the constructive criticism and we are very happy that the manuscript is judged of a general interest in the field.

Major points:

1. *One main concern is the markers used for labeling different subclasses of type I neurons in a number of experimental characterizations. In Fig. 1 and Fig. S1 (quantification of cell number), Fig. 4 (electrophysiological recording), and Fig. 5 (afferent labeling), the authors used CR (CALB2) to label Ia and Ic neurons. However, according to the results shown in Fig. 1f, Calb2 was not only expressed in Ia and Ic neurons but also showed considerable expression in Ib neurons. How could the authors be sure that the CR+ cells only contained Ia and Ic neurons but not Ib neurons?*

We understand the concern of the reviewer when comparing gene expression data and immunostainings. Indeed, antibodies against CR protein was used to label Ia and Ic neurons

specifically while the transcript could be observed, although at much lower levels (more than 6 times less), in the Ib population. This discrepancy between transcript and protein expression is however not unusual, as the presence of mRNA cannot predict the protein expression and marker usage. A recent example of this is the $TrkA^+$ noradrenergic population of sympathetic neurons, which are not labelled by Ret antibody as a marker, while some do express *Ret* at a transcriptional level, though at a lower level (Furlan *et al*., Nat. Neurosci., 2016). Moreover, in cochlea sections, the total absence of peripherin (II marker) or *Lypd1* (Ib marker) expression in CR positive neurons confirm the specificity of CR for the Ia/Ic population (Fig. 1g and Supplementary Figure 1). This has been repeated on 5-6 sections of each region of the cochlea (base, mid and apex), and on cochlea from at least 4 animals, with always identical results. We also conducted co-labelling experiments for *Rxrg* (Ic and II marker) and *Lypd1* (Ib marker) and could never observe any co-localization between the two markers (Supplementary Figure 4b).

Also, in Fig. 5, the authors used Pou4f1 as a marker for Ib neurons. Based on Fig. 1f, this gene was also expressed at a considerable level in Ia neurons. Again, how did the authors ensure labeling specificity? Fig. 5b showed that 95% of Pou4f1+ neurons were Lypd1+ (an Ib neuron-specific marker). However, based on the image shown, less than 50% of Lypd+ neurons were Pou4f1+.

For the *Pou4f1* expression (coding for Brn3a), in reference to Fig. 1 for its expression and Fig. 4 for its usage for the tracing experiment, we took advantage of the fact that *Pou4f1* is 5 times less expressed in Ia than in Ib neurons. While the antibody for Brn3a marks both Ia and Ib populations, limiting the recombination efficiency with only one injection of tamoxifen does limit the recombination specifically to the Ib neurons, where levels of Cre expression should reflect levels of *Pou4f1* (while 5 injections are necessary for high efficiency in other sensory neurons, O'Donovan *et al*., J Exp Med, 2014). This was confirmed by co-labelling with *Lypd1* (an Ib marker) and CR (Ia/Ic marker) as shown in Fig. 4b. Only 5% of RFP positive cells were CR positive, but the majority was *Lypd1* positive, confirming the sparse labelling strategy for targeting Ib neurons for tracing experiments. This has been now clarified in the main text page and in the Material and Methods section under the subtitle "Tamoxifen-induced sparse labelling".

This suggested that a subpopulation of Ib neurons were not labeled by Pou4f1. Have the authors examined the innervation pattern of this subpopulation?

With one injection of tamoxifen to activate recombination in target cells, Ib neurons are preferentially labelled (94% of RFP^+ type I neurons are Ib neurons), but with an efficiency of about 25% at best in the Ib population (see Materials and Methods sections). This explains why only few Ib neurons are genetically labelled (RFP^+) in Figure 4b. This low efficiency (25%) is unlikely therefore to reflect the existence of distinct populations of Ib neurons, but rather the randomness of the recombination events within high *Pou4f1* expressing Ib neurons. Moreover, the relatively homogeneous expression of *Pou4f1* in the Ib neurons would argue against the existence of 2 populations of Ib neurons based on *Pou4f1* expression. As mentioned above, more details for this strategy are now added in the main text and in the Materials and Methods section.

In general, why not use markers that were restricted to a single subtype, e.g. Calb1 for Ia, Lypd1 for Ib, and Trim54 for Ic?

In reference to the Figure 4, to analyze the innervation pattern, we would need markers, and thus antibodies that can be used to label nerve endings below the hair cells, or neuron-specific reporter mouse lines. We have used *Pou4f1*^{CreERT2} for sparse labeling of the Ib population. We have tested numerous antibodies for Calb1 and for Lypd1, but none worked on cochlea tissue. Trim54 is a RING finger protein and is expected to be detected in nuclei, and would therefore not be a good marker for labelling nerve endings underneath the HCs. New mouse lines specific for each subclass of type I cochlear neurons are thus needed in the future.

For quantification purpose, we have analyzed both Runx1 and Brn3a expression (new Supplementary Fig. 1f), together with CR and peripherin expression on sections (Fig. 1 and Suppl. Fig. 1). We also confirmed these results with that of *Lypd1* expression (obtained by RNAscope), and observed similar proportions of cell types in all conditions (Fig. 1g and 1i and Supplementary Fig. 1e and f). Of note, although *in situ* hybridization with RNAscope technology shows very high specificity of cell type, its efficiency is rarely maximal. Thus, *Calb1*, *Rxrg* or *Pcdh20* cannot be used for absolute quantification.

2. *For the validation of subtype markers presented in Fig. 1g and Fig. 6c, no quantification was provided. The authors should quantify the overlap between markers that were predicted to label the same or different subtypes.*

We apologize to the reviewer if the information on co-localization was not clearly stated in the main text or figure legends. This has now been added in the legend of Fig. 1g and 6c and of Supplementary Figure 4b. Note that amongst all markers presented for each subclass, quantification has been performed for *Pou4f1* (Brn3a, for Ia/Ib), Runx1 (for Ia/Ib), *Lypd1* (for Ib) and calretinin (CR, for Ia/Ic)) and *Etv4* (for type II) (about 90% of peripherin$^+$ type II neurons express *Etv4* at P3 basal region). CR, *Lypd1* and peripherin never co-localized. Similarly, Brn3a never co-localized with peripherin and only co-localized with one subpopulation of CR$^+$ neurons (Suppl. Fig. 1f). *Scn4b,* which is type I specific, never co-localized with peripherin (Suppl. Fig. 4a and 4b). *Calb1* was only expressed in one subpopulation of CR positive cells, and never in *Lypd1* positive cells or in *Pcdh20* (Ic and II specific) positive cells. *Cacna1g* was only expressed in peripherin positive type II neurons. *Grm8* was only expressed in *Lypd1* positive Ib neurons. *Etv4* was only expressed in peripherin positive cells at P3. These data are in Fig. 1g and 6c and in Supplementary Fig. 1 and 4.

3. *The authors proposed that the different expression levels of the K+ channel Kv3.2 in different Ib neuron subpopulations may contribute to their different electrophysiological properties (UA vs. MA type). This is an interesting hypothesis and should be directly tested by post-hoc immunostaining of Kv3.2 (as the authors have done for CR) to examine whether there is indeed a correlation between Kv3.2 expression level and whether an Ib neuron is UA or MA type.*

We acknowledge that this is an important point. For this, we have conducted a large number of experiments and tested various antibodies for $K_v3.2$. We have used various antibodies from Alomone lab and Sigma, though no immunostaining for $K_v3.2$ could be found in the litterature. We varied the conditions: concentration, temperature, decalcification or not, PFA or MetOH fixation, fixation duration, antigen retrieval, on sections, whole mount or in vitro (dissociated cells), incubation time, and age of animals. Unfortunately, while our control antibodies showed positive staining (peripherin, βIII-tub, CR etc.), we could not observe any positive staining for $K_v3.2$ in all conditions tested. We

do not think however that it reflects a lack of sufficient expression levels, as other markers show similar levels in the RNAseq and can be used to label cell type.

If the reviewer would feel inappropriate or inadequate to present the Kv3.2 data as it is without functional validation, the data on $K_v3.2$ could be removed from the present study as we think that its absence would not change the essence of the paper. Otherwise, we have stressed in the main text the need to validate this observation functionally in the future. This has been added within the main text, at the end of the second last paragraph of the electrophy. section of the Results. "To the best of our analysis, *Kcnc2* was the only candidate gene in our neurotransmission-related gene dataset to show this contrasted expression in type Ib neurons. In view of the importance of K_v3 channels in the regulation of the firing properties of neurons (Kaczmarek and Zhang, 2017), it will be interesting to assess *in vitro* and *in vivo* the role of $K_v3.2$ in the physiology of type Ib neurons and in hearing."

Minor points:

1. *For the gene set enrichment analysis, It should be indicated what genes were used as background for the analysis; only the expressed genes should be used. Also, it should be indicated whether the p values have been corrected for multiple testing.*

We used differentially expressed genes as input for Gene Set Enrichment Analysis, so no background genes was needed here. Also, all p-values have been corrected using Benjamini & Hochberg False Discovery Rate (FDR) correction. This information has been added in the Materials and Methods section, at the end of the paragraph *GSEA Visualized by Network*:
"Significant GO terms (p-value<0.05, corrected using Benjamini & Hochberg False Discovery Rate (FDR) correction) were used as input for Cytoscape's plugin EnrichmentMap to generate a network where mutually overlapping gene sets cluster together. Following parameters were used: P-value cutoff 0.001, FDR Q-value cutoff 0.05, similarity cutoff with Jaccard coefficient 0.25."

2. *Fig. S3b and S3c were mislabeled.*

We apologize for this mistake which has been corrected.

3. *In Fig. 6i and Fig. S5f, do the plots show all genes or only gene families related to neurotransmission? Were the p values corrected for multiple testing? These were not clear from the text or figure legends.*

Those plots in Fig. 6i show only gene families related to neurotransmission. We have corrected the Figure legend of Fig. 6i by adding this information. Current *p*-values have not been corrected for multiple testing. We had done multiple testing correction for Ia, Ib and Ic neurons but not for type II neurons (since there are only 7 type II neurons in our data set), so we decided to treat and show all data the same way. Of note, multiple testing correction does not change data for type I subclasses of neurons (see Fig. below). This information has now been added in the Materials and Methods:
"Comparison between P3 and adult. The average gene expression of SG neuron types at P3 and adult were used to compute Pearson's correlation coefficient. Differentially expressed genes between P3 and adult were identified using Wilcoxon rank sum test. P-value and log fold change of genes were used to compute volcano plot. We have compared the volcano plots obtained using the P-values with

those using the multiplicity adjusted P-values and found no difference. The P-value for all volcano plots are shown in the Figure 6 to keep the consistency between all neuronal subclasses, since the type II subclass has only 7 cells in our dataset, and multiplicity adjusted P-values cannot be applied for this group.”

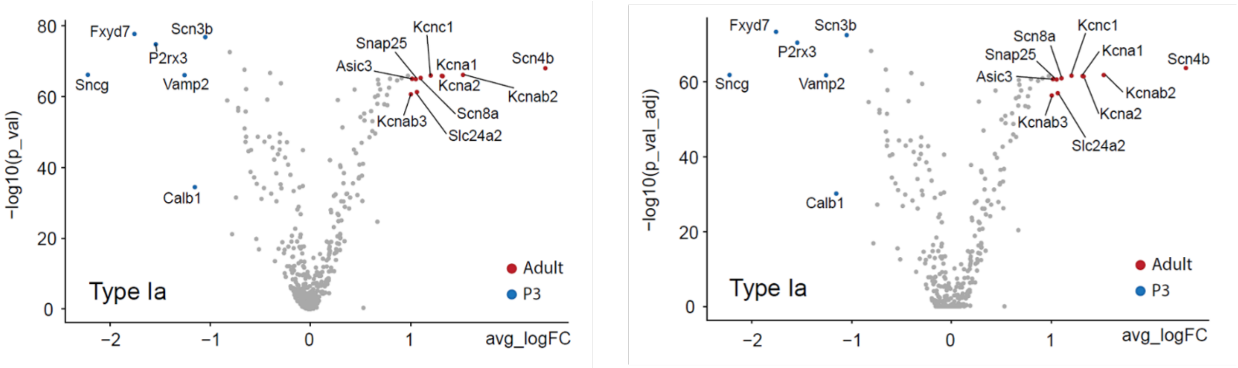

Figure. Comparison between the non-corrected (left) versus the corrected (right) version of the volcano plots for Ia neurons between P3 and adult stage.

4. *In the Abstract, "exhaustive transcriptional catalog" seems an overstatement, given that only hundreds of cells have been profiled. Additional molecular subtypes could be identified when more cells are sampled. Indeed, the electrophysiological experiments in this study suggested that the Ib neurons may be further comprised of subpopulations of different electrophysiological properties.*

This sentence in the abstract has been changed by removing exhaustive.

Reviewer #3

We are glad that the reviewer finds that our manuscript is of high quality and should provide an excellent resource to investigators in the field. Please find below a response to the criticism and an outline on the modifications introduced into the manuscript to fully address the issues on controls and quantifications.

Major concerns:

1. *Some of the most important data points can be found in the violin plots. In the figures shown, the violin plots are far too small. The y axes are compressed, and they are difficult to interpret in a quantitative sense. For all of the violin plots shown, the dots that indicate the individual cells that contributed to the width of the violin are needed. The dots help show the raw data and allow the reader to compare between groups.*

We are glad that the reviewer wishes to ensure high visibility of the data and have now added new panels in the Supplementary Figures 1d and 7a to show the details of each violin plots of the Figures 1 and 6, with the dots representing single cell values.

2. *Most of the histology is limited to the core set of factors that were discovered that define each group. Why not add some in situ hybridization experiments for select factors within those groups? As it stands, Figures 2, 3, and 7 is all data analysis. For example, the authors should show in situ*

hybridization data for one or two of their "functional signatures" genes as proof of concept and show that the cells with high levels of those genes appear at the expected frequency.

We have followed the suggestion of the reviewer and included expression data on functional genes to the existing ones. The new data concerns *Scn4b, Scl17a6*, *Grm8*, *Cacna1g* and *Kcnip2* and have now been added in the Suppl. Figure 4a and 4b. The new data confirm the single cell RNAseq results.

3. *The authors should show some proof that there is no contamination by hair cells into the SGN sorts. The use of PV;Cre brings this possibility. I became concerned about this seeing that VGlut1 and Piezo2 were noted as SGN markers, and these factors are known to be expressed by hair cells. Apparently, the sensory domain is mechanically separated from the SGNs for their sorts, but some evidence showing that the tdTomato population that was used for the sort did not contain hair cells would be beneficial. The presence of Prox1, for example, engenders confidence that the cells are mostly SGNs, but Prox1 is known to be expressed transiently in hair cells (albeit at much earlier stages).*

This is indeed a very important point in our scRNAseq data, which we carefully addressed before analyzing them in details. We regret that this was not clear enough in the first version of our study. The expression in adult neurons of *Prox1,* but also of *Isl1*, *Tubb3* (βIII-tubulin), *Syp* (synaptophysin) and of many genes commonly expressed in neurons and related to neurotransmission and which were in Fig. 3 and Supplementary Fig. 1a together with the dissection procedure give strong confidence on the neuronal identity of the cells we isolated and sequenced. We also originally searched for markers found only in HCs, such as *Pou4f3*, *Atoh1* and *Gfi1*, which we did not find to be expressed in our dataset. To respond to the reviewer's comment, we now have added these data in Supplementary Fig. 1a. Regarding *Vglut1* and *Piezo2*, those are two genes commonly expressed in sensory neurons, such as neurons of the dorsal root ganglia where they are used as specific markers (Oliveira *et al*., Synapse, 2003; Woo *et al*., Nat Neurosci, 2015; Ranade *et al*., Nature, 2014; Zeisel *et al*., bioRxiv, 2018).

4. *For all of these new markers shown by either immunofluorescence or RNAscope, there is no discussion or demonstration of staining specificity. Indeed, the lack of staining in neighboring cells acts as an "internal control," but some information on the epitopes for these factors and the ways that their specificity was ascertained would be helpful, especially for anyone wanting to replicate their findings.*

We agree with the reviewer that the specificity aspect of the RNAscope method has not been stated in the original version of our manuscript. This information has now been added in the Material and Methods section:

"RNA *in situ* hybridization experiments were performed using RNAscope®, an RNA *in situ* hybridization technique described previously (Wang F et al., J Mol Diagn, 2012). Paired double-Z oligonucleotide probes were designed against target RNA using custom software. The RNAscope® Reagent Kit (Advanced Cell Diagnostics, Newark, CA) was used according to the manufacturer's instructions (kit version 1). Frozen fixed tissue sections were prepared according to manufacturer's recommendations. Each sample was quality controlled for RNA integrity with a probe specific to the housekeeping gene *Ppib*. Negative control background staining was evaluated using a probe specific to the bacterial *DapB* gene."

Also, please note that amongst all markers presented for each subclass in Fig. 1g for instance, quantification has been performed for *Pou4f1* (Brn3a), Runx1, *Lypd1* and calretinin (CR) and *Etv4* (Fig. 1i and Suppl. Fig. 1) (about 90% of peripherin^{+} type II neurons express *Etv4* at P3 basal region). CR, *Lypd1* and peripherin never co-localized. Similarly, Brn3a never co-localized with peripherin and only co-localized with one subpopulation (Ia population) of CR^{+} neurons (Supplementary Figure 1f). *Scn4b*, which is type I specific, never co-localized with peripherin (Suppl. Fig. 4a and 4b). *Calb1* was only expressed in some calretinin positive cells, and never in *Lypd1* positive cells or in *Pcdh20* positive cells. *Cacna1g* was only expressed in peripherin positive type II neurons. *Grm8* was only expressed in *Lypd1* positive Ib neurons. *Etv4* was only expressed in peripherin positive cells at P3. These data gives further confidence in the specificity of our stainings. This has now been added in the legend of the Fig. 1g and 6c and of the Supplementary Figure 4b.

5. *All of the significant p-values can be found in the supplementary Excel spreadsheet. This is very unsatisfying. In addition, the colorful squares are very nice and beautifully arranged in the figures, but the reader is left with no sense of the raw data or significance values. I would recommend that the authors devise a way, for at least some of the important genes, to display statistical significance.*

We understand the concern of the reviewer and followed the suggestion. We added new data showing boxplots for the expression of all important genes (categories associated to neuron's identity) (see Suppl. Figure 2, 3 and 8). This indeed provides a more direct comparison of raw expression data of differentially expressed genes between clusters.

6. *For figure 5 on the spatial segregation of the type I SGNs, the authors need to quantify the differences they are describing. This analysis needs to be far more rigorous. As it stands the reader has no reason to be confident in the illustration in 5g. What criteria did the authors use to define "pillar" vs "modiolar?" What neighboring cells were used as landmarks?*

We thank the reviewer for this comment and quantified the data and explained in more details the methods used for the analysis of the neuron projection patterns. These data are added in Figure 5 d-f and Suppl. Fig. 6d and 6e, and text has been added under the subtitle "Quantification of the neuronal fibers:" in the Materials and Methods section.

Minor points:

1. *For the micrographs shown, the ages of the mice should be indicated.*

This has been added in the Figure legends where appropriate

2. *The first sentence of the Abstract needs to be revised. The possessive "brain's" doesn't really fit.*

This has been corrected

REVIEWERS' COMMENTS:

Reviewer #1 (Remarks to the Author):

The authors addressed my concerns/ comments and revised the manuscript to my satisfaction.

I have only one minor comment:
The authors write(line 80): "To identify neuron types in adult SG neurons, a total of 487 TOM+ cells from PVCre;R26TOM cochlea of postnatal stage 17 (P17), P21 and P33 were processed for single-cell transcriptome analysis (Fig. 1a-c).

My suggestion is to include a sentence that explains the genetic labeling strategy and why this particular Cre line was used. Also the authors should define the used abbreviations (e.g Tom+).

Reviewer #2 (Remarks to the Author):

The authors have addressed all of the reviewer's comments. With regard to the Kv3.2 data, it is fine to keep it in the manuscript after adding the statement mentioned in the authors' response letter.
The reviewer now considers the paper to be appropriate for publication in Nature Communication.

Reviewer #3 (Remarks to the Author):

I feel the manuscript has been strengthened by the additions made by the authors, but I am only satisfied partially. It seems that many of the changes/additions made were relegated to the supplementary data section, especially some of the important sets of raw data and statistical values. This ideological difference between me and the authors can be left up to the editors at Nature Communications, but I really feel these things should be displayed in the main figures. In their rebuttal letter, the authors allude to aiming for "high visibility," so why not put these things in the manuscript figures that are most visible to the readership? I also still have concerns about the data in figure 5 (see below, point #4).

Main points:

The violin plots with the dots showing individual cells should be in the main figures, not the supplement. The y-axes in newly added violin plots are not scaled equally and this also needs to be corrected for better direct comparisons between sets.

From my original point #2, I appreciate the additions of Scn4b, etc., but again think these should be added to the main manuscript figures, not the supplement. For these new images

in supplemental 4a and b, the authors have strangely chosen to use a combination of dark blue, light blue, and teal. All three of these channels have a blue contribution >0. Assuming they are using RGB (red-green-blue) mode, this isn’t appropriate for a merged image because it is ambiguous. The authors need to change this or show separated channels.

In my point #5 from the original review, I had suggested that the authors display some statistical values in the main manuscript figures. They didn’t do this and instead added box plots to the supplement. I can live with this, but would ask that the authors, again, scale the y-axes equally.

For figure 5 (my original comment #6), the authors have only provided quantification data for CR -- why? The purpose is to compare the subtypes of fibers and their synaptic locations in a quantitative sense. So, this analysis in its current form is of limited value. Also, the description of the quantification method is lackluster and needs to have much more detail. Based on the description provided, as a reader, I don’t have much confidence in the data because I don’t know how they designated the different IHC regions. They need to better describe how they imaged the cells, what landmarks they used, and how they dealt with variability in how the tissue samples were oriented.

Response to reviewers

REVIEWERS' COMMENTS:

Reviewer #1 (Remarks to the Author):

The authors addressed my concerns/ comments and revised the manuscript to my satisfaction.

We are very glad that the reviewer is satisfied with our revised version; please find below our response to her/his remaining minor comments.

I have only one minor comment:
The authors write(line 80): "To identify neuron types in adult SG neurons, a total of 487 TOM+ cells from PVCre;R26TOM cochlea of postnatal stage 17 (P17), P21 and P33 were processed for single-cell transcriptome analysis (Fig. 1a-c).

My suggestion is to include a sentence that explains the genetic labeling strategy and why this particular Cre line was used. Also the authors should define the used abbreviations (e.g Tom+).

A sentence explaining the genetic strategy has now been added line 82-83. Also, TOM+ is now explained line 80.

Reviewer #2 (Remarks to the Author):

The authors have addressed all of the reviewer's comments. With regard to the Kv3.2 data, it is fine to keep it in the manuscript after adding the statement mentioned in the authors' response letter. The reviewer now considers the paper to be appropriate for publication in Nature Communication.

We are very glad to read that the reviewer finds our revised version acceptable for publication. The Statement in question (and written in the previous response to reviewers) was in fact added in the manuscript, lines 237-241.

Reviewer #3 (Remarks to the Author):

I feel the manuscript has been strengthened by the additions made by the authors, but I am only satisfied partially. It seems that many of the changes/additions made were relegated to the supplementary data section, especially some of the important sets of raw data and statistical values. This ideological difference between me and the authors can be left up to the editors at Nature Communications, but I really feel these things should be displayed in the main figures. In their rebuttal letter, the authors allude to aiming for “high visibility,” so why not put these things in the manuscript figures that are most visible to the readership? I also still have concerns about the data in figure 5 (see below, point #4).

We are glad that the reviewer finds our revised version to be strengthened. Please find below our response to her/his last remaining comments.

Main points:

The violin plots with the dots showing individual cells should be in the main figures, not the supplement. The y-axes in newly added violin plots are not scaled equally and this also needs to be corrected for better direct comparisons between sets.

The message provided by the violin plots that are depicted in the main figures is to show differences in the expression of specific marker genes between newly identified populations of neurons. This main message needs to be clearly highlighted for the reader. Moreover, the important comparison is within genes and between groups, and this contrast is shown more clearly if the vertical scale for each gene is adjusted individually. The absolute levels of gene expression are affected by many factors, both technical and biological, and do not correspond directly to levels of the corresponding protein. Therefore, comparison between genes is less informative, and we prefer to highlight the differences that exist between groups. For these reasons, magnification of the violin plots, with the dots, is depicted in the supplementary file. But an invitation to read the detailed plots in the Supplementary file is now added to the Figure legend of Fig. 1 and 6.

Together with adding new Supplementary files, we have created an online searching database that will be accessible directly on our lab website https://ki.se/en/neuro/lallemend-laboratory *through a specific link (that will be added upon acceptance of the manuscript). This database will allow anyone to search for any gene expression in the 4 subclasses of SG neurons, both in postnatal (P3) and adult samples. Gene expression will be depicted as violin plots, with the choice of showing individual dots, the mean and standard deviation (see example in the figure beside for Lypd1 and Calb2 genes in adult and P3 samples, respectively).*

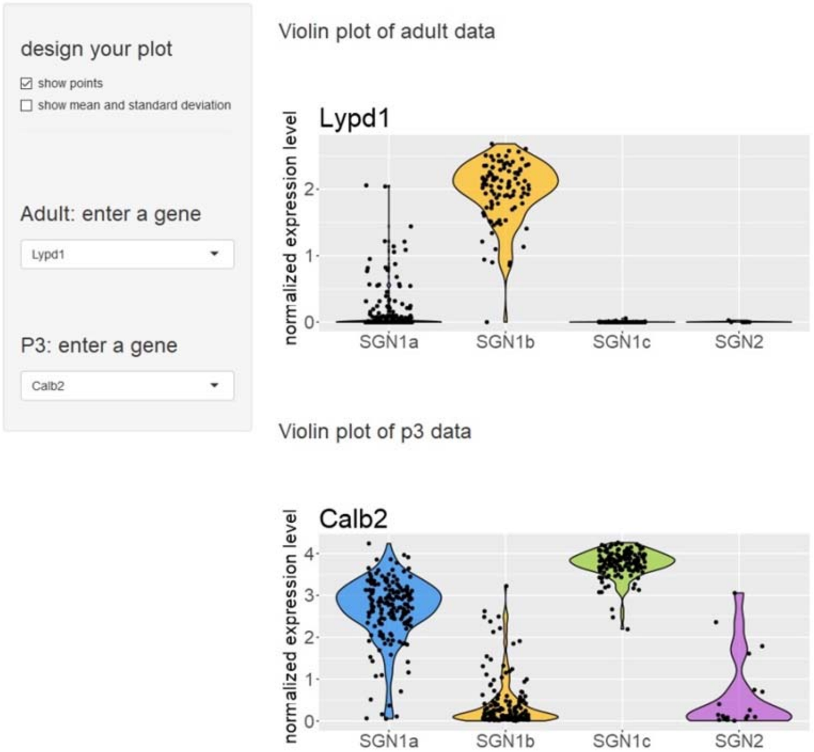

From my original point #2, I appreciate the additions of Scn4b, etc., but again think these should be added to the main manuscript figures, not the supplement. For these new images in supplemental 4a and b, the authors have strangely chosen to use a combination of dark blue, light blue, and teal. All three of these channels have a blue contribution >0. Assuming they are using RGB (red-green-blue) mode, this isn't appropriate for a merged image because it is ambiguous. The authors need to change this or show separated channels.

The Figure 3 of the manuscript is providing a detailed overview of all differentially expressed genes in SG neuron types that are associated with neurotransmission, together with a scheme of the connectivity of SG neurons and of the differentially expressed neurotransmission-related genes at the pre- and post-synaptic regions of the neurons. The comment from the first round of revision to confirm in situ the expression of more functional genes was highly appropriate, and new results had been added in the Suppl. Figure 4a,b in the previous version. These new results indeed strengthened our RNAseq data shown in Figure 3. We however believe that they should not be depicted in the main figures and should remain in the Supplementary file as they serve to control and confirm the RNAseq, and do not participate directly to the understanding and to the main message of the Figure 3.

For the Supplementary Fig. 4b, the color code has been changed according to the reviewer's suggestion.

In my point #5 from the original review, I had suggested that the authors display some statistical values in the main manuscript figures. They didn't do this and instead added box plots to the supplement. I can live with this, but would ask that the authors, again, scale the y-axes equally.

Statistics had indeed been added to the data, as Supplementary file, in our previous version. Similar to our response to the first comment, the important comparison is within genes and between groups, and this contrast is shown more clearly if the vertical scale for each gene is adjusted individually. Comparison between genes is less informative, and we prefer to highlight the differences that exist between groups, which are in the main Figure. Any details of the raw data can be found in the Supplementary Figures, Supplementary data sheets, in our searching database (soon online on our lab website) and in GEO.

For figure 5 (my original comment #6), the authors have only provided quantification data for CR -- why? The purpose is to compare the subtypes of fibers and their synaptic locations in a quantitative sense. So, this analysis in its current form is of limited value. Also, the description of the quantification method is lackluster and needs to have much more detail. Based on the description provided, as a reader, I don't have much confidence in the data because I don't know how they designated the different IHC regions. They need to better describe how they imaged the cells, what landmarks they used, and how they dealt with variability in how the tissue samples were oriented.

We apologize for not having been clearer in the description of the new data presented in the Figure 5 on the quantification of the innervation of type I neurons with IHCs. This has now been added in the main text, lines 271-274: "A quantitative analysis of CR^+ (only in Ia/Ic neurons) versus PV^+ (in all neurons) fibers underneath the IHCs in cochlea whole mount of WT mice further confirmed the specific projection of Ib neurons to the modiolar and of the Ia/Ic neurons to the pillar (Ia/Ic) side of the IHCs (Fig. 5d-f, Supplementary Fig. 6d-e)."

Also, a more detailed description of the method used to orient the organ of Corti and define the IHC regions is now added in the Method section, under Quantification of the neuronal fibers (lines 555-564, underlined is the new text): "Whole mount cochlea were stained by using calretinin (CR) to target Ia/c fibers and parvalbumin (PV) for all type I SG neuron fibers <u>underneath the IHCs</u>. For each cochlea, 3 areas with 7 IHCs per area from mid-basal levels were imaged <u>(Z-stacks through the organ of Corti)</u> and analyzed. <u>The organ of Corti was 3D reconstructed, and the baso-apical orientation of the IHCs was assessed by using the stereocilia of the apical region of the IHCs as landmarks. The IHC side facing the OHCs was considered as the pillar side, and the IHC side facing</u>

the SG neurons, the modiolar side. Each Z-stack section was analyzed using ImageJ, first by defining the area of interest (below the IHCs). Automated selection of the immunostained area for either PV or CR positive fibers was done using "analyze particle" in ImageJ and measured."
